# Epigenetic switching as a strategy for quick adaptation while attenuating biochemical noise

**DOI:** 10.1371/journal.pcbi.1007364

**Published:** 2019-10-28

**Authors:** Mariana Gómez-Schiavon, Nicolas E. Buchler

**Affiliations:** 1 Program in Computational Biology & Bioinformatics, Duke University, Durham, North Carolina, United States of America; 2 Center for Genomic & Computational Biology, Duke University, Durham, North Carolina, United States of America; 3 Department of Biology, Duke University, Durham, North Carolina, United States of America; 4 Department of Physics, Duke University, Durham, North Carolina, United States of America; New York University, UNITED STATES

## Abstract

Epigenetic switches are bistable, molecular systems built from self-reinforcing feedback loops that can spontaneously switch between heritable phenotypes in the absence of DNA mutation. It has been hypothesized that epigenetic switches first evolved as a mechanism of bet-hedging and adaptation, but the evolutionary trajectories and conditions by which an epigenetic switch can outcompete adaptation through genetic mutation remain unknown. Here, we used computer simulations to evolve a mechanistic, biophysical model of a self-activating genetic circuit, which can both adapt genetically through mutation and exhibit epigenetic switching. We evolved these genetic circuits under a fluctuating environment that alternatively selected for low and high protein expression levels. In all tested conditions, the population first evolved by genetic mutation towards a region of genotypes where genetic adaptation can occur faster after each environmental transition. Once in this region, the self-activating genetic circuit can exhibit epigenetic switching, which starts competing with genetic adaptation. We show a trade-off between either minimizing the adaptation time or increasing the robustness of the phenotype to biochemical noise. Epigenetic switching was superior in a fast fluctuating environment because it adapted faster than genetic mutation after an environmental transition, while still attenuating the effect of biochemical noise on the phenotype. Conversely, genetic adaptation was favored in a slowly fluctuating environment because it maximized the phenotypic robustness to biochemical noise during the constant environment between transitions, even if this resulted in slower adaptation. This simple trade-off predicts the conditions and trajectories under which an epigenetic switch evolved to outcompete genetic adaptation, shedding light on possible mechanisms by which bet-hedging strategies might emerge and persist in natural populations.

## Author summary

Epigenetic switches regulate cell fate decisions during development in multicellular organisms, but their origin predates multicellularity because they are found in viruses, bacteria, and unicellular eukaryotes. It has been suggested that epigenetic switches first evolved as a mechanism of bet-hedging and adaptation to fluctuating environments. To discern the evolutionary pressures that select for epigenetic switches, we used computer simulations to evolve a mechanistic, biophysical model of a self-activating genetic circuit, which can both adapt genetically and exhibit epigenetic switching. Unlike laboratory evolution experiments, this *in silico* experiment was run many times over a range of evolutionary parameters (population size, selection pressure, mutation step-size, fluctuation frequency) and different model assumptions to uncover statistical regularities in the evolutionary trajectories. Using this computational approach, we could elucidate simple principles that predict the conditions that favor adaptation by epigenetic switching over genetic mutation in a fluctuating environment.

## Introduction

Populations often need to adapt to environmental changes in order to survive. Classic models of genetic adaptation presume that random genetic mutations generate novel phenotypes that can be selected by the environment [1]. Under some circumstances, e.g. small populations or fast fluctuating environments, the spontaneous appearance of a beneficial mutation might be too infrequent and, thus, some organisms may induce genetic variation to accelerate adaptation to a new environment. Examples include adaptive mutation, where genetic variation occurs in response to the environment [2], directed mutation, where non-random and useful mutations are induced [3], and cryptic variation, where non-neutral mutations accumulate and are buffered without phenotypic consequences until the environment changes [4].

Alternatively, many organisms have evolved biochemical networks that directly sense environmental cues and induce phenotypic responses to the new environment (known as “phenotypic plasticity”). The frequency of environmental change, the accuracy of cues, the penalty of non-adaptation, as well as the cost of producing the sensing machinery will determine the advantage of phenotypic plasticity [5, 6].

A third option is to generate spontaneous phenotypic variation (i.e. not in response to the environment) but restrict this variation to specific phenotypic states. This is possible through the evolution of epigenetic switches, where multiple stable phenotypic states (e.g. level of gene expression) are possible in a genetically identical population and stochastic transitions between these states occur without genetic mutations [7]. This corresponds to the original definition of “epigenetics” by Waddington [8], and epigenetic switches have been proposed as one mechanism of bet-hedging to deal with fluctuating environments [9]. Epigenetic switches can emerge through self-reinforcing feedback loops in biochemical or genetic networks, and the epigenetic phenotypes are heritable from mother to daughter cells [10]. Examples of epigenetic switches include heritable gene regulation by self-reinforcing transcription factor activity, DNA methylation, chromatin modification, non-coding RNAs, and prions [11]. Spontaneous transitions between epigenetic states (an “epimutation”) can occur due to biochemical fluctuations and noisy gene expression, where the epimutation rate is usually much faster than genetic mutation [12]. Epimutation generates phenotypic diversity and quick adaptation when one of the new states is favored in the changed environment; on the other hand, these frequent epimutations impose a cost in the population when the environment remains unchanged. As such, epigenetic switches represent a bet-hedging strategy, and an alternative to slower genetic adaptation and costly phenotypic plasticity. Epigenetic switches have been shown to occur in natural populations [13], and to evolve as an adaptation to fluctuating selection during laboratory evolution of microbes [14, 15]. Nevertheless, the specific evolutionary conditions that lead to the emergence and selection of epigenetic switches remain to be elucidated.

The advantage of phenotypic plasticity over epigenetic switches in fluctuating environments has been extensively studied using mathematical models and simulations [5, 16–36]. Phenotypic plasticity was almost always advantageous when the associated cost was low (i.e. small induction delays, environmental cues reliably switch cells to optimal adapted phenotypes, small metabolic burden of biochemical network). In conditions where phenotypic plasticity was disfavored or not available, the authors showed that an epigenetic switch conferred an advantage when the spontaneous epimutation rates matched the environmental fluctuation rate and when selection pressures on fit/unfit phenotypes were symmetric between the two different environments (see S1 Appendix for details).

Many of the models used in these studies did not include genetic adaptation through mutation, a competing process that occurs in all organisms. Genetic mutations can modify biophysical parameters of protein-DNA and protein-protein interactions, and thus affect the levels and dynamics of gene expression. To better understand the evolutionary dynamics of an epigenetic switch in a fluctuating environment, we used computer simulation to evolve a self-activating gene in a fluctuating environment where genetic mutations changed biophysical parameters and, thus, modified gene expression (i.e. phenotype). A self-activating gene is the simplest mechanistic model of a genetic network that can simultaneously adapt genetically and exhibit epigenetic switching. In a specific region of the biophysical parameter space, the expression of a self-activating gene can be bistable (e.g. two stable states) and biochemical noise could spontaneously induce a transition between these two stable states (e.g. epimutation).

We evaluated the evolutionary dynamics of this simple circuit over a broad range of evolutionary parameters (population size, selection pressure, mutation step-size, environmental fluctuation frequency) and tested different model assumptions. In all tested conditions, populations initially evolved by genetic mutation to genotypes (parameter space) with high nonlinearity, even in the absence of biochemical noise, in agreement with previous work [28, 37]. We developed a simple predictive model to show that the circuit’s genetic potential under fluctuating selection increases in this region of parameter space. Briefly, an adapted genotype has a large genetic potential if a short mutational path exists to another genotype that is well-adapted to the alternative environment, thus facilitating the genetic adaptation after an environmental transition [38]. Once in the high-nonlinearity regime, we show that a trade-off exists where the adaptation time after each environmental transition –*adaptation potential*– and the cost imposed by the biochemical noise –*noise load*– cannot be simultaneously optimized. Moreover, the balance of this trade-off depends on the specific nonlinearity value (as measured by the Hill coefficient *n*_*H*_). By tracking genotypes and individual lineages that persisted –with or without mutations– across generations, we show that epigenetic switching was in fact selected in fast fluctuating environments, where the benefit of the adaptation potential is maximized. As the environmental fluctuation frequency decreases, genetic adaptation was favored and selected even higher nonlinearity values optimizing the noise load. Also, lineage tracking allowed us to identify a hybrid strategy where a bistable population would adapt genetically, which can optimally balance the described trade-off when the mutation step-size (*M*) was small in slowly fluctuating environments. Finally, by estimating the expected noise load, and the associated epimutation probability, for each genotype, we show that the evolutionary advantage of epigenetic switching is that it allows for quick adaptation while still attenuating the biochemical noise level.

## Results

**Fig 1:**
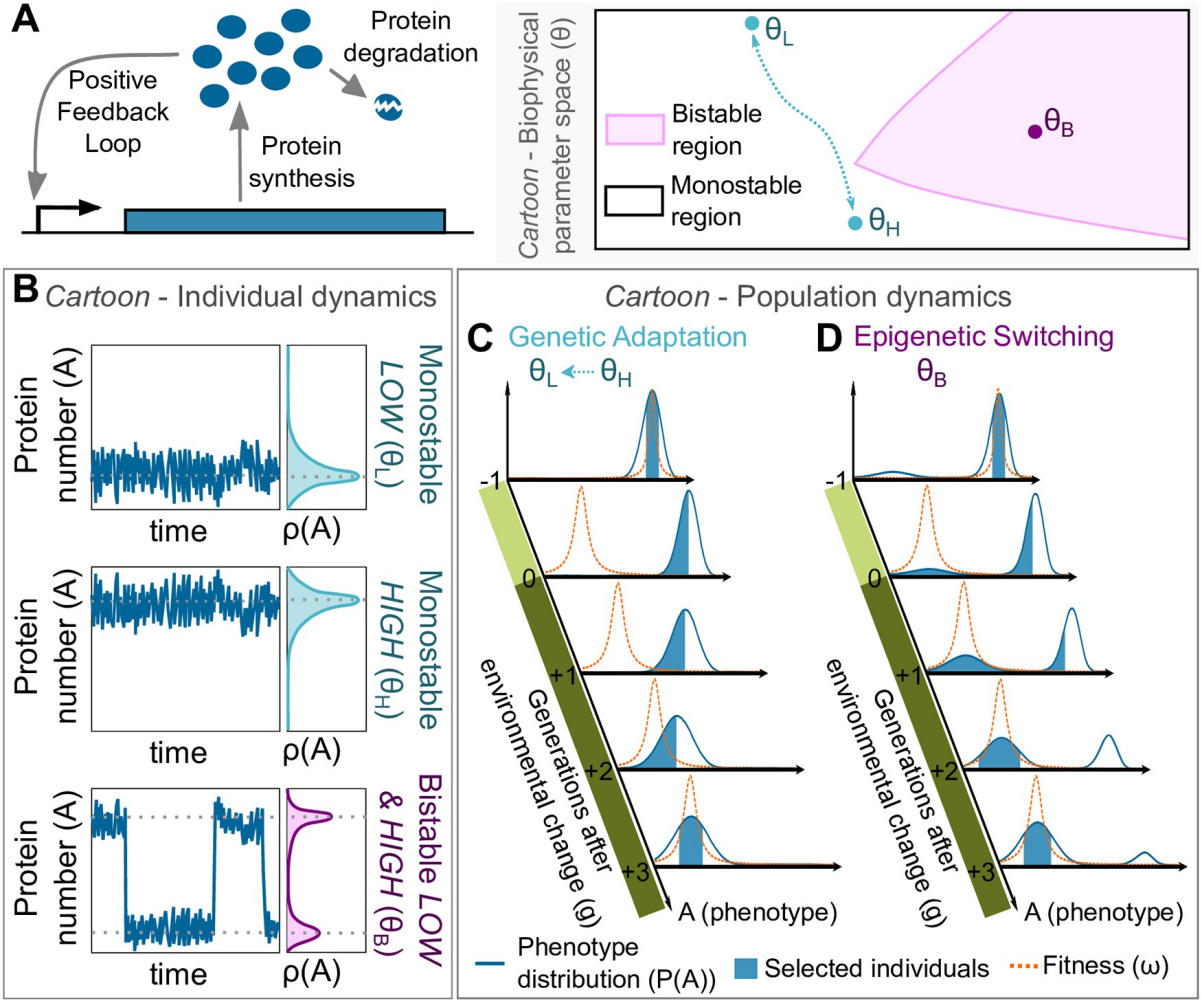
Genetic adaptation and epigenetic switching of a self-activating gene. (A) Diagram of a self-activating gene that considers two biochemical events: protein synthesis with a rate that increases with number of proteins *A* (i.e. positive feedback loop) and protein degradation. On the right, a cartoon of the biophysical parameter space or genotypes (*θ*) of this gene circuit with two characteristic regions: monostable (white) and bistable (pink) phenotypes. In the monostable region, a genotype (*θ*_*L*_ or *θ*_*H*_) might be optimal in either one environment (e.g. LOW protein numbers) or the other (e.g. HIGH protein numbers). Genetic mutations are required to change from one solution the other (blue arrow). In the bistable region, a single genotype (*θ*_*B*_) can display two different phenotypes with each phenotype potentially optimal in both environments. (B) Cartoon of the protein number (*A*) dynamics in an individual cell with each of the genotypes described in (A). Monostable genotypes (*θ*_*L*_ and *θ*_*H*_) exhibit a unimodal distribution of protein expression (*ρ*(*A*)), whereas a bistable genotype (*θ*_*B*_) exhibits a bimodal distribution having spontaneous transitions between phenotypic states over time (i.e. epimutations) triggered by stochastic gene expression. Cartoon of the population dynamics using (C) genetic adaptation or (D) epigenetic switching to adapt after an environmental change. The fitness score function (*ω*; orange dashed line) and the phenotype distribution of the population (*P*(*A*); blue line) are shown for each generation (*g*), and the fraction of the population expected to be selected in the next generation (i.e. individuals with higher fitness scores) are highlighted (blue area). The environment changes from selecting HIGH protein numbers (light green) to select LOW protein numbers (dark green).

A self-activating gene can display a unimodal distribution (monostable) or a bimodal distribution of protein number (bistable), depending on the underlying biophysical parameters (Fig 1 and S1 Fig). The spontaneous transitions (i.e. *epimutation*) between bistable phenotypes are driven by stochastic gene expression. Genetic mutations change the biophysical parameters (i.e. *genotype*) and will modify both the distribution of protein number (i.e. *phenotype*, *ρ*(*A*)) and the stochastic transition rate between bistable phenotypes. Two qualitatively different strategies of evolutionary adaptation could emerge from a self-activating gene. The population could evolve from one monostable distribution to another by mutating its biophysical parameters after each environmental change (i.e. *genetic adaptation*). Alternatively, the population could reside at a bistable solution where each bimodal state is optimal in one of the environments and epimutations from one bistable state to another occur over time without an underlying genetic mutation (i.e. *epigenetic switching*). In both cases, the phenotypic distribution of the population *P*(*A*) expands each generation due to gene expression noise and mutations, but natural selection keeps it centered on the optimal protein number as determined by the fitness function of the current environment. After an environmental change, the fitness function changes and the tail of the phenotypic distribution with higher fitness will be selected every generation. In the case of genetic adaptation, this selection will gradually shift the population towards the new optimal phenotype through the accumulation of new mutations until the population is well-adapted again. The speed of genetic adaptation depends on the rate of arrival of fitter mutations, as well as the selection pressure. When the population applies epigenetic switching, the phenotypic noise also includes epimutations, which increase the maladapted fraction in the population (epimutational load). However, after the environment and the fitness function change, the “epimutated” individuals rapidly overtake the population, quickly shifting the distribution to the new optimal value. Thus, genetic adaptation and epigenetic switching can directly compete as two strategies of adaptation to a fluctuating environment.

### Stochastic dynamics of a self-activating gene

For simplicity, we considered two biochemical events (protein synthesis or degradation) that either increase or decrease the number of proteins *A* by one molecule:

$$\begin{array}{ccc} \text{Protein}\;\text{synthesis}: & A & \overset{f(A)}{\to }A+1 \end{array}$$ (1)

$$\begin{array}{ccc} \text{Protein}\;\text{degradation}: & A & \overset{\gamma \cdot A}{\to }A-1 \end{array}$$ (2)

The synthesis rate *f*(*A*), which describes the probability per unit time that protein synthesis occurs and that *A* is increased by one, is a nonlinear function of activator *A*:

$$\begin{array}{c} f\left(A\right)=k\cdot (\alpha +\left(1-\alpha \right)\frac{A^{n_{H}}}{A^{n_{H}}+K_{D}^{n_{H}}}) \end{array}$$ (3)

where *k* (number of proteins/unit time) represent the maximum synthesis rate, *α* is the basal synthesis rate relative to *k*, *K*_*D*_ (number of proteins) is related to the protein-DNA dissociation constant, and *n*_*H*_ is the degree of molecular cooperativity (i.e. Hill coefficient). The degradation rate *γ* ⋅ *A*, which describes the probability per unit time that protein degradation occurs and that *A* is decreased by one, is a linear function of activator *A* where *γ* (1/unit time) is the protein degradation rate constant. With no loss of generality, we defined the unit of time as *τ* = *t* ⋅ *γ*, which allows us to substitute time *t* with a time-dimensionless variable (see Methods). We used the Gillespie algorithm, a kinetic Monte Carlo method that explicitly simulates the probabilistic dynamics of a defined set of biochemical events [39], to simulate the stochastic dynamics of our gene circuit (see Methods).

### Evolutionary model

**Fig 2:**
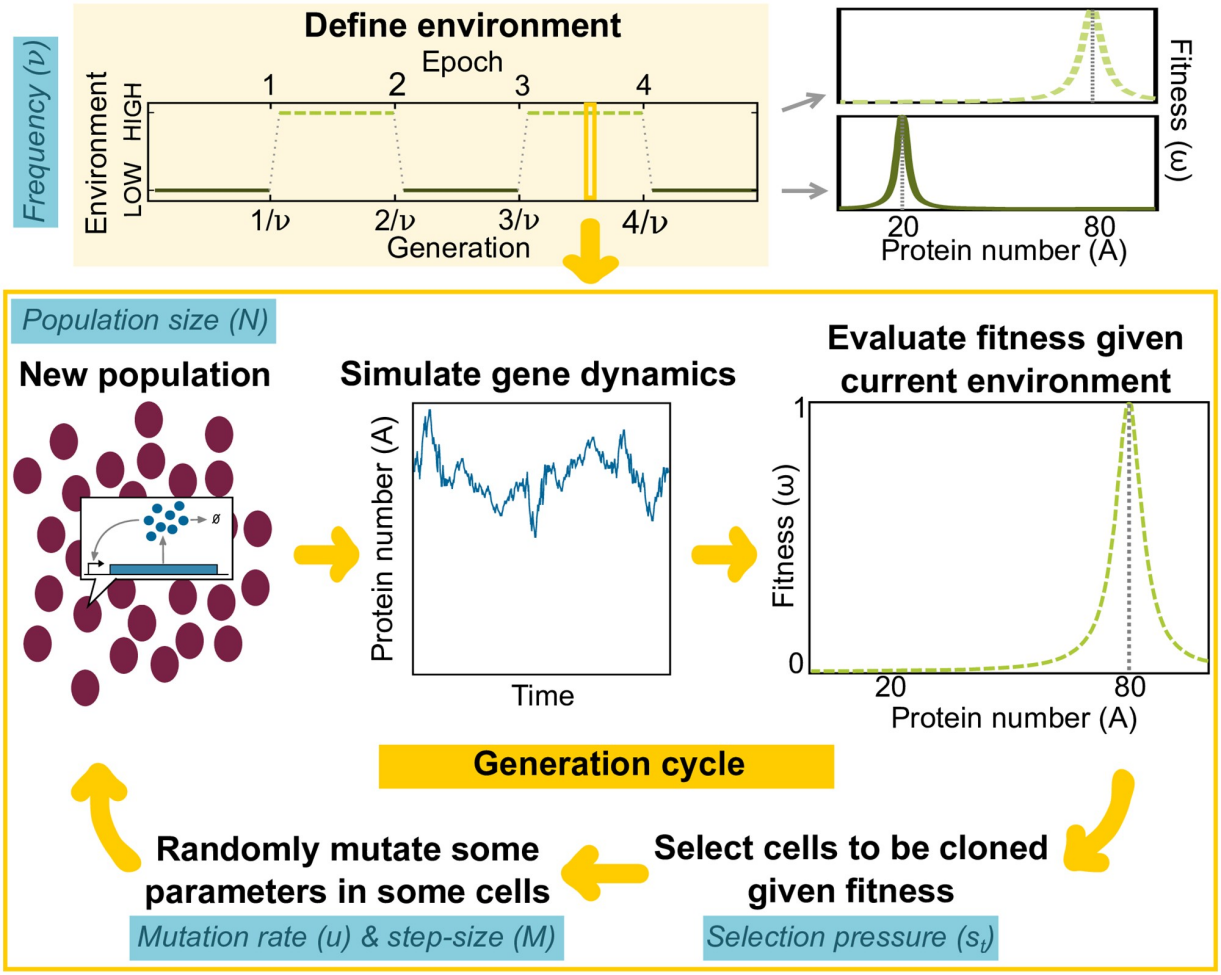
Evolutionary model. The environment fluctuates periodically with frequency *ν*. The total number of generations spent in a constant environment (epoch) has the same length (1/*ν*) and each environment (HIGH or LOW) selects for a different distribution of protein levels (phenotypes). Each generation, we simulated the stochastic protein dynamics of a self-activating gene in each cell across a population of size *N*. At the end of each simulation, the population phenotypes varied because gene expression is stochastic and because cells can have different underlying biochemical parameters (genotypes). The current environment in each generation assigned a fitness (*ω*_*i*_) to each cell (*i*) based on its final protein level. We used tournament selection (where *s*_*t*_ determines the strength of selection) to determine the next generation of cells according to their fitness. Each cell in the next generation was mutated with probability *u*, where the current set of biophysical parameters were multiplied or divided up to a maximum step-size of *M*.

For simplicity, we evolved a haploid, asexual population with non-overlapping generations in a fluctuating environment (Fig 2). The underlying biophysical parameters depend on protein stability, protein-protein, protein-RNA, and protein-DNA interactions, which can change through genetic mutations. For simplicity, we allowed mutations on the maximum synthesis rate (*k*), Hill coefficient (*n*_*H*_) and DNA dissociation constant (*K*_*D*_) during our evolutionary simulations, while keeping basal activity (*α* = 0.25) and degradation rate (rescaled, *γ* = 1) fixed. The set of variable parameters {*k*, *n*_*H*_, *K*_*D*_} are the genotype (*θ*). The environment fluctuated periodically with frequency *ν* between LOW (selects for optimal phenotype *A*^(*L*)^ = 20 proteins) and HIGH (selects for optimal phenotype *A*^(*H*)^ = 80 proteins). The number of generations spent in a constant environment (“epoch”) was equal to 1/*ν* generations. The selective environment switched to the alternative state at the end of an epoch. Starting from an isogenic population, we ran each evolutionary simulation for 10,000 generations. We implemented the following algorithm each generation:

1. Simulate stochastic gene dynamics of a self-activating gene in each cell given its biophysical parameters (i.e. genotype) and initial protein level inherited from its parent in the previous generation for 4 units of time (the cell “life span”).
2. Evaluate the fitness of each cell *i* based on the protein level at the end of its life span (*A*_*i*_, phenotype). The individual fitness function (*ω*_*i*_) is:
  $$\begin{array}{c} \omega_{i}^{\left(E\right)}\left(A_{i}\right)=\frac{\epsilon^{2}}{\epsilon^{2}+{\left(A_{i}-A^{\left(E\right)}\right)}^{2}} \end{array}$$ (4)
  where *E* = {*L*, *H*} is the current environment, *A*^(*E*)^ is the optimal phenotype for each environment, and *ϵ*^2^ = 0.2 ⋅ *A*^(*E*)^ is the width of the Lorentzian function. We define the population fitness (*w*) as the average $\Sigma_{i=1}^{N}\omega_{i}^{\left(E\right)}\left(A_{i}\right)/N$ over all cells.
3. Select the next generation using tournament selection [40, 41], where *s*_*t*_ cells are chosen randomly from the population. The cell with highest fitness within the chosen cohort is cloned into the new population. This “tournament” is repeated *N* times with replacement to create a new population. The tournament size (*s*_*t*_) modulates the selection pressure, where small *s*_*t*_ is weak selection (e.g. for *s*_*t*_ = 1, there is no selection pressure and only genetic drift because any randomly selected individual is the tournament winner). Increasing *s*_*t*_ leads to stronger selection and a faster selective sweep of fitter cells (e.g. for *s*_*t*_ = *N*, only the fittest individual in the entire population will be cloned into the next generation).
4. Allow random mutations with a fixed probability (*u*) in each cloned cell. If a mutation occurs, the parameter values in the cell genotype are updated as follows:
  $$\begin{array}{ccc} k^{\prime } & \leftarrow & k\cdot M^{r\sqrt{1-\phi_{1}^{2}}\cos\left(\phi_{2}\right)} \end{array}$$ (5)
  $$\begin{array}{ccc} n_{H}^{\prime } & \leftarrow & n_{H}\cdot M^{r\sqrt{1-\phi_{1}^{2}}\sin\left(\phi_{2}\right)} \end{array}$$ (6)
  $$\begin{array}{ccc} K_{D}^{\prime } & \leftarrow & K_{D}\cdot M^{r\phi_{1}} \end{array}$$ (7)
  where *M* is the maximum fold change (mutation step-size), *r* ∼ *U*(0, 1), *ϕ*_1_ ∼ *U*(−1, 1), and *ϕ*_2_ ∼ *U*(0, 2*π*) are uniformly distributed random values between 0 and 1, -1 and 1, and 0 and 2*π*, respectively (see Methods). This spherically symmetric 3D mutation scheme permits co-variation in biophysical parameters in a single mutational step, with the “distance” to the parental genotype uniformly distributed (in the logarithmic scale) in the range defined by the mutation step-size. Unlike additive mutation, multiplicative mutation better reflects how mutations affect thermodynamics of protein stability, protein-DNA and protein-protein interactions [42, 43]. All mutated parameters were constrained to lie within a physiological range that is typical for a bacterial transcription factor (see Methods).
5. If the evolutionary simulation is at the end of an epoch, then change to other environment; otherwise keep the same environment. Return to Step 1 to simulate next generation.

### Populations adapt to the fluctuating environment

The evolutionary model was simulated over a range of population size (*N* = [100, 10000]), selection pressure (*s*_*t*_ = [3, 250]), environmental fluctuation frequency (*ν* = [0.01, 0.1]), mutation rate (*u* = [0.01, 0.1]), and mutation step-size (*M* = [1.1, 5]). We restricted these parameters to regions where our simulations were feasible and where epigenetic switching and genetic adaptation were competitive with one another. For example, we only considered *uN* ≥ 1; otherwise, mutations were too infrequent for genetic adaptation to compete with epigenetic switching. We verified that our results presented below were robust to alternative assumptions, such as different models of selection, non-periodic environmental fluctuations, a Moran model of reproduction, and alternative mutation schemes (see Methods).

To control for the possibility that bistability could be selected for reasons other than epigenetic switching [28], we also ran a parallel deterministic simulation (CONTROL) of the expression of the auto-activating gene in Step 1 (see Methods). Given that there is no biochemical noise in our CONTROL simulations, cells in the bistable region display hysteresis and stay in the stable state closest to the inherited parental state and never stochastically switch to the other state (i.e. no epigenetic switching).

**Fig 3:**
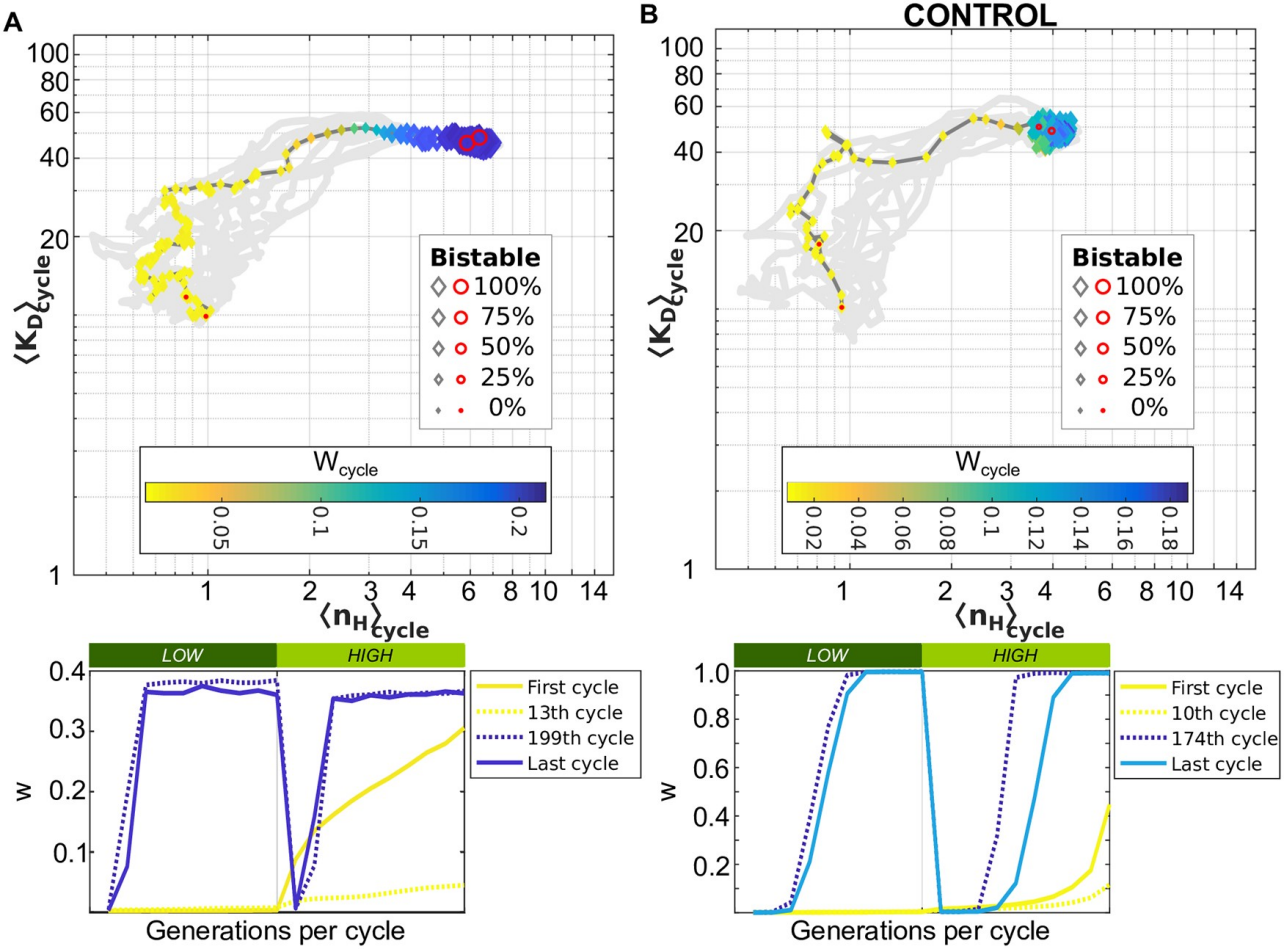
Evolutionary trajectory of a population adapting to a fast fluctuating environment displays selection for high nonlinearity. The initial population started from a non-optimal genotype (*θ*_0_) where *k* = 80, *n*_*H*_ = 1, *K*_*D*_ = 10 with evolutionary parameters *N* = 10000, *ν* = 0.1, *s*_*t*_ = 40, *u* = 0.03, and *M* = 1.1 both under (A) the original model and (B) in the absence of biochemical noise (*CONTROL*). The average *K*_*D*_ (〈*K_D_*〉_cycle_) versus the average Hill coefficient (〈n_H_〉_cycle_) are shown for each environmental cycle simulated, colored given the geometric mean of the population fitness per environmental cycle ($W_{\text{cycle}}=\sqrt[{2/\nu }]{\Pi_{g=\{\text{cycle}\}}w_{g}}$), and the marker size corresponds to the average percentage of bistable genotypes in the population (see legend). The traces of other nine replicas are shown in gray as a reference. Each cycle spans a LOW (dark green) and HIGH (light green) epoch and there are 500 environmental cycles over 10,000 generations for each of these simulations. The population fitness *w* per generation for the first and last cycles, as well as the cycle with the minimum and maximum *W*_cycle_ are shown in the bottom for comparison, and colored according to their *W*_cycle_ value. In both simulations (A-B), the population evolved to higher nonlinearity values 〈n_H_〉_cycle_ and *W*_cycle_.

All populations started with a monostable genotype *θ*_0_ that was adapted to one environment but not the other. These populations all evolved to a higher fitness solution. Initially, populations had a slightly higher geometric mean fitness per cycle $W_{\text{cycle}}=\sqrt[{2/\nu }]{\Pi_{g=\{\text{cycle}\}}w_{g}}$ because it accrued benefits by being adapted to the HIGH environment despite being maladapted to the LOW environment (Fig 3A). We consider geometric mean fitness per cycle rather than fitness per generation (*w*) because it better reflects the long-term growth of fitter phenotypes in natural populations [44], but analogous results are obtained considering the arithmetic mean instead. During the LOW epoch that follows the HIGH epoch, the populations shifted towards higher *w*^(*L*)^ values. The epoch was too short and mutation too weak for the populations to perfectly adapt to the new environment before it changed again. In all cases, we observed that the evolutionary dynamics in early epochs were dominated by noisy genetic adaptation of a population maladapted to at least one of the environments, even if this implied decreasing *W*_cycle_. The “no-response” behavior, i.e. being adapted to one environment and “ignoring” the alternative state, is not a stable solution for this system. Consistent with previous work, this illustrates the importance of considering the full population dynamics in the adaptation process and not only the long-term average fitness [45, 46].

Sub-optimal populations eventually increased the nonlinearity in gene expression 〈*n_H_*〉_cycle_. For example, in a fast-fluctuating environment (*ν* = 0.1) with small mutation step size (*M* = 1.1), the population evolved to a bistable genotype *θ*_*B*_ that used epigenetic switching (Fig 3A); nevertheless, high *n*_*H*_ values appear in the population before transitioning from a monostable to a bistable genotype (e.g. 〈*n*_*H*_〉 ∈ [2, 3.3] and less than 10% bistable individuals per generation). In this case, the bistable genotype was a global optimum in *W*_cycle_ and adaptation occurred in a few generations after each environmental change due to epigenetic switching. We verified that this bistable genotype was globally optimal by re-running evolutionary simulations for different initial genotypes (*θ*_0_) and for more generations. The CONTROL simulations, where epigenetic switching was not available, also evolved high nonlinearity in gene expression 〈*n_H_*〉_cycle_, but kept monostable genotypes (Fig 3B).

### Selecting for genetic potential

Meyers *et al*. [38] have shown that populations in variable environments can stably evolve to higher *genetic potential*, i.e. genotypes with a higher sensitivity to mutations. To explore this possibility in our model, we took advantage of the defined mechanistic model, where for a given steady state A_*_ (i.e. deterministic solution of the gene expression system, $\frac{dA_{*}}{dt}=f\left(A_{*}\right)-\gamma A_{*}=0$), and each value of *K*_*D*_ and *n*_*H*_, the synthesis rate value k_*_ is uniquely defined as follows:

$$\begin{array}{c} k_{*}=\frac{\gamma A_{*}\left(A_{*}^{n_{H}}+K_{D}^{n_{H}}\right)}{A_{*}^{n_{H}}+\alpha K_{D}^{n_{H}}} \end{array}$$ (8)

**Fig 4:**
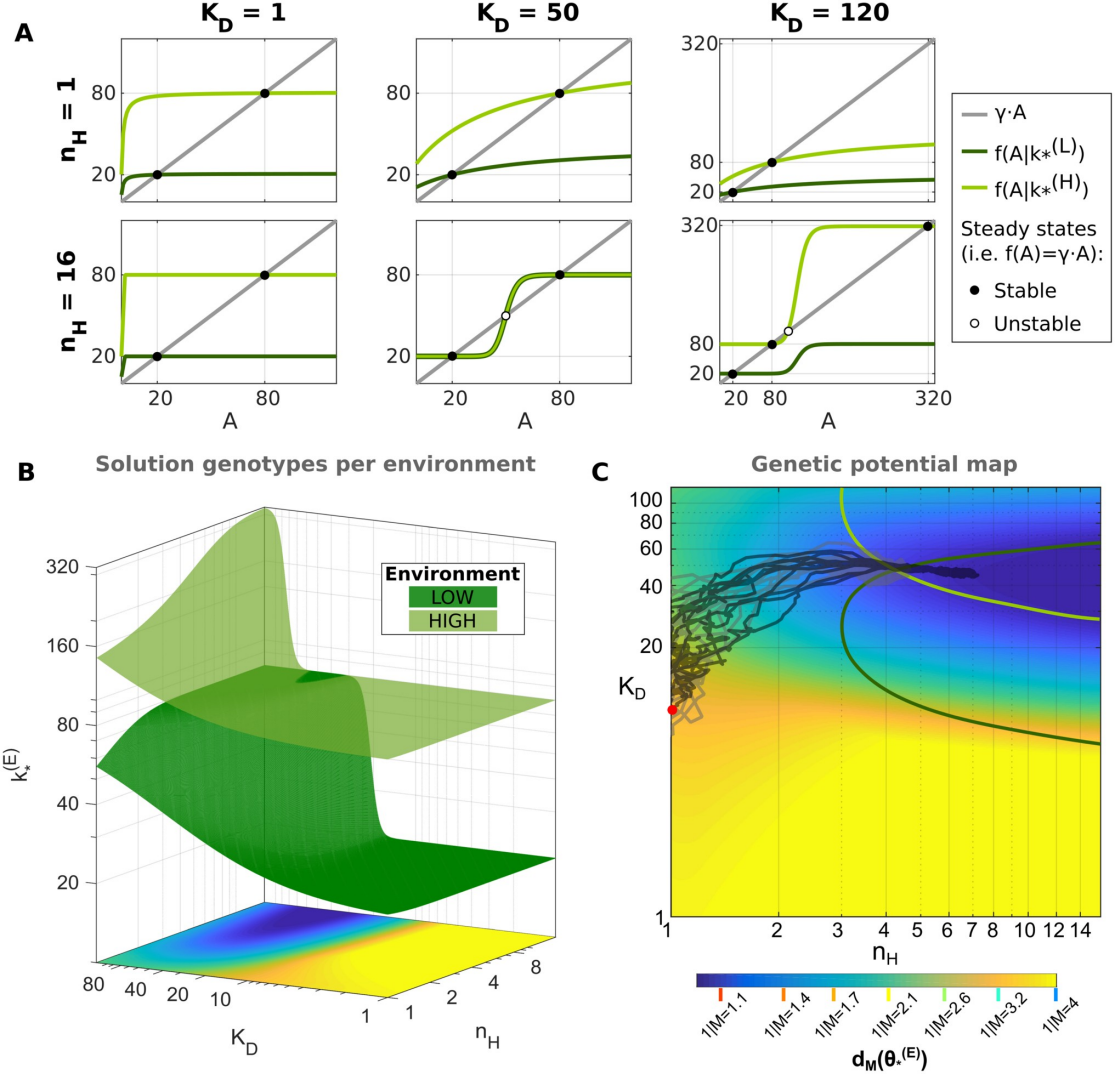
Genetic potential increases around the bistable region at high nonlinearity. (A) The *f*(*A*) function for some solution genotypes are shown (see row and column titles) exemplifying cases where (1) both $\theta_{*}^{\left(L\right)}$ and $\theta_{*}^{\left(H\right)}$ are monostable (i.e. the associated *f*(*A*) and *γ* ⋅ *A* intersect only once; all examples with *n*_*H*_ = 1, and {*n*_*H*_ = 16, *K*_*D*_ = 1}), (2) a bistable solution genotype with $k_{*}^{\left(L\right)}\approx k_{*}^{\left(H\right)}$ ({*n*_*H*_ = 16, *K*_*D*_ = 50}, *f*(*A*) and *γ* ⋅ *A* intersect both in *A* = 20 and *A* = 80, with an unstable steady state between these steady states), and (3) only $\theta_{*}^{\left(H\right)}$ is bistable, and not a solution for the *LOW* environment ({*n*_*H*_ = 16, *K*_*D*_ = 120}). (B) Solution genotypes $\theta_{*}^{\left(E\right)}$ per environment were calculated (Eq 8). (C) The maximum genetic potential for different values of *K*_*D*_ and *n*_*H*_ (i.e. $\text{min}\left(d_{M}\left(\theta_{*}^{\left(L\right)}\right),d_{M}\left(\theta_{*}^{\left(H\right)}\right)\right){|}_{\left\{n_{H},K_{D}\right\}}$) is shown in the colormap. As reference, the bistable solution genotypes for each environment are delimited by the green lines: $\theta_{*}^{\left(L\right)}$ as dark green, and $\theta_{*}^{\left(H\right)}$ as light green. Additionally, we show as an example the population average *K*_*D*_ and *n*_*H*_ per cycle for ten simulations over 10000 generations with *k* = 80, *n*_*H*_ = 1, and *K*_*D*_ = 10 as the initial genotype (red circle, *θ*_0_), and evolutionary parameters *N* = 10000, *u* = 0.03, *M* = 1.1, *ν* = 0.10, and *s*_*t*_ = 40 (black lines), and the equivalent CONTROL simulations (i.e. without biochemical noise; gray lines). The colorbar shows the one-mutation distance corresponding to each value of *M*.

Then, the optimal synthesis rate $k_{*}^{\left(E\right)}$ can be calculated for each environment *E* with an optimal phenotype $A_{*}^{\left(E\right)}$ (Fig 4A and 4B).

For each solution genotype, $\theta_{*}^{\left(E\right)}=\left\{k_{*}^{\left(E\right)},n_{H},K_{D}\right\}$, we calculate the *genetic potential* as the minimum genetic distance to adapt after an environmental transition, i.e. to reach any solution genotype in the alternative environment:

$$\begin{array}{c} d_{M}\left(\theta_{*}^{\left(L\right)}\right)={\text{min}}_{j\in \theta }(∥{\text{log}}_{M}\left(\theta_{*}^{\left(L\right)}\right)-{\text{log}}_{M}\left(\theta_{*,j}^{\left(H\right)}\right)∥) \end{array}$$ (9)

$$\begin{array}{c} d_{M}\left(\theta_{*}^{\left(H\right)}\right)={\text{min}}_{j\in \theta }(∥{\text{log}}_{M}\left(\theta_{*}^{\left(H\right)}\right)-{\text{log}}_{M}\left(\theta_{*,j}^{\left(L\right)}\right)∥) \end{array}$$ (10)

When $\theta_{*}^{\left(L\right)}\approx \theta_{*}^{\left(H\right)}$ (i.e. $d_{M}\left(\theta_{*}^{\left(E\right)}\right)\approx 0$ for both environments), bistability arises and adaptation can occur with a noise-induced epimutation (Fig 4). If there is no noise (e.g. deterministic CONTROL), then the bistable region exhibits hysteresis and the system can only switch when genetic adaptation forces the genotype to leave the bistable region (usually through *K*_*D*_ mutations).

We observed that starting from an arbitrary initial genotype *θ*_0_, populations always migrated to a region of higher genetic potential, until the solution to the alternative state is reachable in just one genetic mutation, if this is available. This occurred regardless of the specific evolutionary conditions (e.g. selection pressure *s*_*t*_ or environmental fluctuation frequency *ν*), or the absence of noise (i.e. CONTROL simulations). In the gene expression model described here (Eqs (1) and (2)), the genetic potential increases around the bistable region, which occurs at high nonlinearity *n*_*H*_ (Fig 4C). We also analyzed the double negative feedback loop (“toggle switch”), an alternative system with the ability to display bistability and epigenetic switching. The genetic potential of the toggle switch also increases with higher nonlinearity and particularly around the bistable region (S2 Fig). This supports the generality of our observations, as each of these systems (positive versus double negative feedback loops) produce different bifurcation types [47]. These results show that the initial selection for high genetic potential can drive the population to a genotypic space where bistability is available, suggesting a potential mechanism for the initial emergence of epigenetic switches in fluctuating environments.

Notably, the region where the alternative solution is reachable in one genetic mutation depends directly on the mutation step-size *M* (Eqs (9) and (10); Fig 4C). Congruently, CONTROL simulations with higher *M* values showed a higher diversity, with no obvious selection pressure once the population is inside this optimal genetic potential region (S3 Fig). Nevertheless, simulations with biochemical noise resulted in more constraint genotype distributions regardless of the specific mutation step-size.

### Trade-off between adaptation time and phenotypic robustness

**Fig 5:**
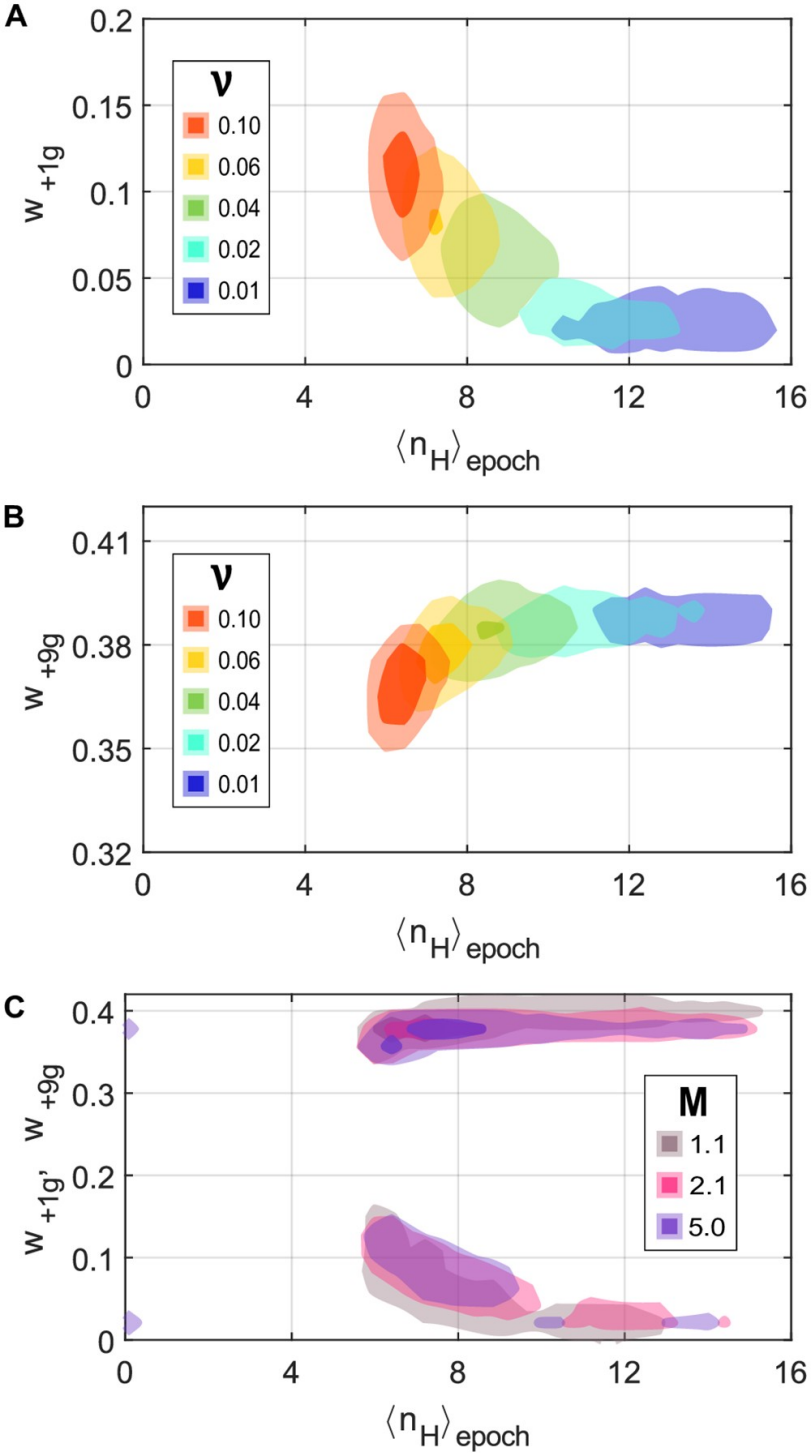
Trade-off between minimizing adaptation time and maximizing phenotypic robustness correlates with nonlinearity. Contour plot of the bi-variate histogram of the average Hill coefficient per epoch 〈*n_H_*〉_epoch_ and the population fitness at the (A) first generation *w*_+1*g*_ and (B) ninth generation *w*_+9*g*_ after an environmental transition are shown grouped by the environmental fluctuation frequency (*ν*). (C) An analogous plot but grouped by the mutation step-size value (*M*; three example values) shows that the observed trend is related to the *ν* value. In all cases, the last 100 epochs of ten independent replicas were considered, each simulation running for 10,000 generations with evolutionary parameters *N* = 10000, *s*_*t*_ = 40, *u* = 0.03 and *k* = 80, *n*_*H*_ = 6 and *K*_*D*_ = 45 as the initial genotype (*θ*_1_; this initial genotype sped up evolutionary simulations by being closer to final selected genotypes in all simulations). In each group, the shade area shows the bins with at least 1% of cases, and at least 5% of cases in the darker area.

In all evolutionary conditions tested here, the optimal *W*_cycle_ was obtained in the high nonlinearity regime. Nevertheless, the average Hill coefficient increased as the environmental fluctuation frequency decreased (Fig 5; S3 Fig). In order to explore the evolutionary advantage of the distinct Hill coefficient values here, we took a closer look to the population fitness.

The population fitness is always low and maladapted when the environment first changes. Selection favors those genotypes which produce phenotypes that better match the selection pressure in the new environment. In this first phase (the “adaptation phase”), the population fitness one generation after each environmental change (*w*_+1*g*_) display higher values for faster environmental fluctuations (i.e. large *ν*; Fig 5A). However, all populations eventually reach a higher fitness and become adapted to the new environment. In this second phase (the “constant phase”), purifying selection maintains the optimal phenotype against perturbations from gene expression noise, mutations, and/or epimutation. The population fitness nine generations after each environmental change (*w*_+9*g*_) display higher values for slowly fluctuating environments (i.e. small *ν*; Fig 5B). The population spends proportionally more time in the constant phase as the epoch length increases (i.e. *ν* decreases), which favors those strategies with robust phenotypes (i.e. minimize frequent, maladapted phenotypes that arise from biochemical noise and epigenetic switching). Noteworthy, higher mutation step size (*M*) favors a slightly higher *w*_+1*g*_, while slightly decreasing *w*_+9*g*_, but with very similar distributions between groups and no obvious correlation with the selected Hill coefficient (*n*_*H*_; Fig 5C).

These results indicate a trade-off in the evolutionary process between minimizing the adaptation time during the adaptation phase and increasing the robustness of the phenotype during the constant phase. Further, the distribution of Hill coefficient values (〈*n_H_*〉_epoch_) as a function of epoch length suggests that natural selection tunes this trade-off via this biophysical parameter. We additionally corroborated the relationship between Hill coefficient value and the expected fitness in an infinite, isogenic population (**E**[*w*^(*E*)^]); we observed that in the region of interest in the biophysical parameter space (*k* ≈ 80), **E**[*w*^(*E*)^] increases as *n*_*H*_ increases regardless of the value of *K*_*D*_ (S4 Fig). In the following section, we explore the relationship between this fitness trade-off and the selected adaptation strategy.

### Lineage analysis shows the selection of epigenetic switching in fast fluctuating environments

In order to determine the selected adaptation strategy for each evolutionary condition, it was informative to analyze the genealogy (i.e. lineages) of each population (see Methods). We tracked the evolutionary history of each individual in the population to identify those lineages that persisted with or without mutations over one full environmental cycle (i.e. LOW epoch + HIGH epoch; S5 Fig). More than one lineage can persist over a cycle, but fewer than expected from coalescent theory because our population is evolving under selection and faces a selective sweep at each environmental transition [48]. If a particular adaptation strategy is successful, then we expect those lineages using that strategy to have a higher fitness and a larger number of progeny. The weight of each persisting lineage is proportional to the number of progeny at the end of the cycle.

**Fig 6:**
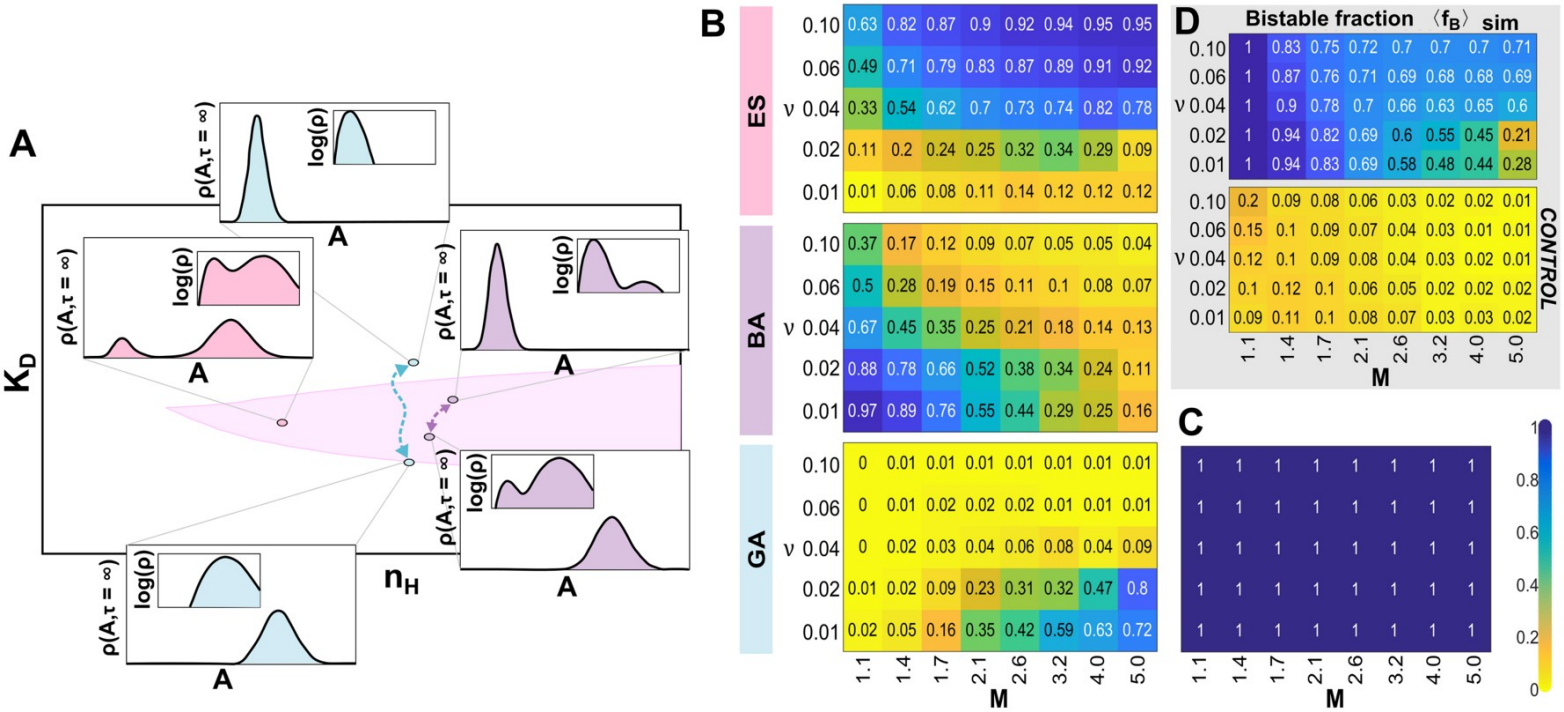
Each adaptation strategy is favored under different evolutionary conditions and the transition between selected strategies is gradual. (A) Illustration of epigenetic switching (ES), bistable adaptation (BA), and genetic adaptation (GA) strategies and underlying genotypes with fixed synthesis rate (*k* = 80). The bistable region of genotypic space is highlighted in pink. The phenotype stationary distribution *ρ*(*A*, *τ* = ∞) for each genotype (*θ*) is shown in the inset, both in linear and logarithmic scale. BA is bistable, as seen in logarithmic scale, but appears effectively monostable in linear scale. This arises because *K*_*D*_ evolves each epoch to favor one mode over the other by decreasing the relative rates of epigenetic switching between the largest and smallest mode. (B) Each colormap shows the fraction of parental lineages using specific adaptation strategy (ES, BA, or GA) averaged over all cycles and ten independent replica simulations for the corresponding mutation step-size (*M*) and environmental fluctuation frequency (*ν*). Each simulation ran for 10,000 generations with evolutionary parameters *N* = 10000, *s*_*t*_ = 40, *u* = 0.03 and *k* = 80, *n*_*H*_ = 6 and *K*_*D*_ = 45 as the initial genotype (*θ*_1_). (C) Results of the CONTROL simulations where gene expression dynamics are deterministic and no stochastic epigenetic switching can occur. All lineages exhibited GA and neither bistable strategy (ES or BA) was ever selected. (D) The corresponding bistable fraction (〈*f_B_*〉_sim_) averaged over all cycles and ten independent replica simulations for the stochastic simulations (top) and deterministic CONTROL (bottom; same simulations than B-C, respectively).

An epigenetic switch can adapt with no mutation; thus, lineages with a bistable genotype and no mutations during a cycle were classified as following an *epigenetic switching* (ES) strategy. On the other hand, those lineages that had at least one monostable genotype and accumulated mutations during a cycle were classified as following a *genetic adaptation* (GA) strategy. Lineages with only bistable genotypes that accumulated mutations during a cycle were classified as following a hybrid *bistable adaptation* (BA) strategy. Although some of these mutations can be neutral, we found that most of them modulated the DNA dissociation constant (*K*_*D*_) and directly affected the rate of epigenetic switching (i.e. epimutation; Fig 6A). Thus, the mutation can be adaptive in the hybrid BA strategy, although the circuit remained bistable and ultimately adapted through epigenetic switching.

Our lineage analysis demonstrated two propositions: (1) epigenetic switching (ES) effectively allows for adaptation to fluctuating environments, outcompeting genetic adaptation, with lineages persisting for multiple environmental cycles without mutations (S5 Fig); and (2) distinct strategies were favored in different evolutionary conditions (Fig 6B). Consistently with previous works, ES was the preferred strategy when the environment fluctuated frequently (i.e. high *ν* values); in slowly fluctuating environments, GA was favored if the mutation step-size (*M*) was large enough, otherwise BA was the dominant adaptation strategy. These trends persisted over a range of different evolutionary parameters, although the boundaries shifted. For example, increasing the selection pressure (*s*_*t*_) or the mutation rate (*u*) shifted boundaries to favor GA, whereas increasing population size (*N*) favored ES (S6 Fig).

Noteworthy, the fraction of bistable genotypes (〈*f_B_*〉_sim_) as a function of evolutionary parameters did not reflect the favored adaptation strategy (Fig 6D). The 〈*f_B_*〉_sim_ never fell to zero even when GA was the optimal lineage strategy (e.g. low *ν*, high *M*). These results arise because of the increased seeding of genetic mutants (which is facilitated by higher *M*) from the monostable to bistable subpopulation. Conversely, we expect more cases of neutral or nearly neutral mutations in the bistable region for smaller mutational step-sizes (*M*). It is exactly in this regime where the fraction of ES parental lineages decreased whereas BA increased. However, most lineages displaying BA in this regime also persisted into the next cycle without accumulating any mutations and, thus, automatically switched to ES (S7 Fig).

The dominant adaptation strategy trend (Fig 6B) highly correlates with the observed fitness trade-off (Fig 5). Moreover, we observed a mixture of strategies across lineages, and the transition between preferred adaptation strategies as a function of *ν* and *M* was gradual. This is expected if the described trade-off is the driving selection force. If each adaptation strategy favors distinct aspects of the trade-off, whenever these fitness costs have similar values, genetic drift will dominate during the selection process. As expected, the simulations in regimes with co-dominant strategies showed high temporal variation in the fraction of adaptation strategies each evolutionary cycle (S7 Fig).

Finally, in our CONTROL simulations with deterministic dynamics (where no stochastic epigenetic switching can occur even if the system is bistable), none of the parental lineages exhibit ES or BA (Fig 6C). This corroborates that the selection of ES as adaptation strategy requires the presence of biochemical noise. In the following section, we show that, in addition of generating epimutations, biochemical noise is also an important fitness component which must be optimized.

### The role of the noise load

**Fig 7:**
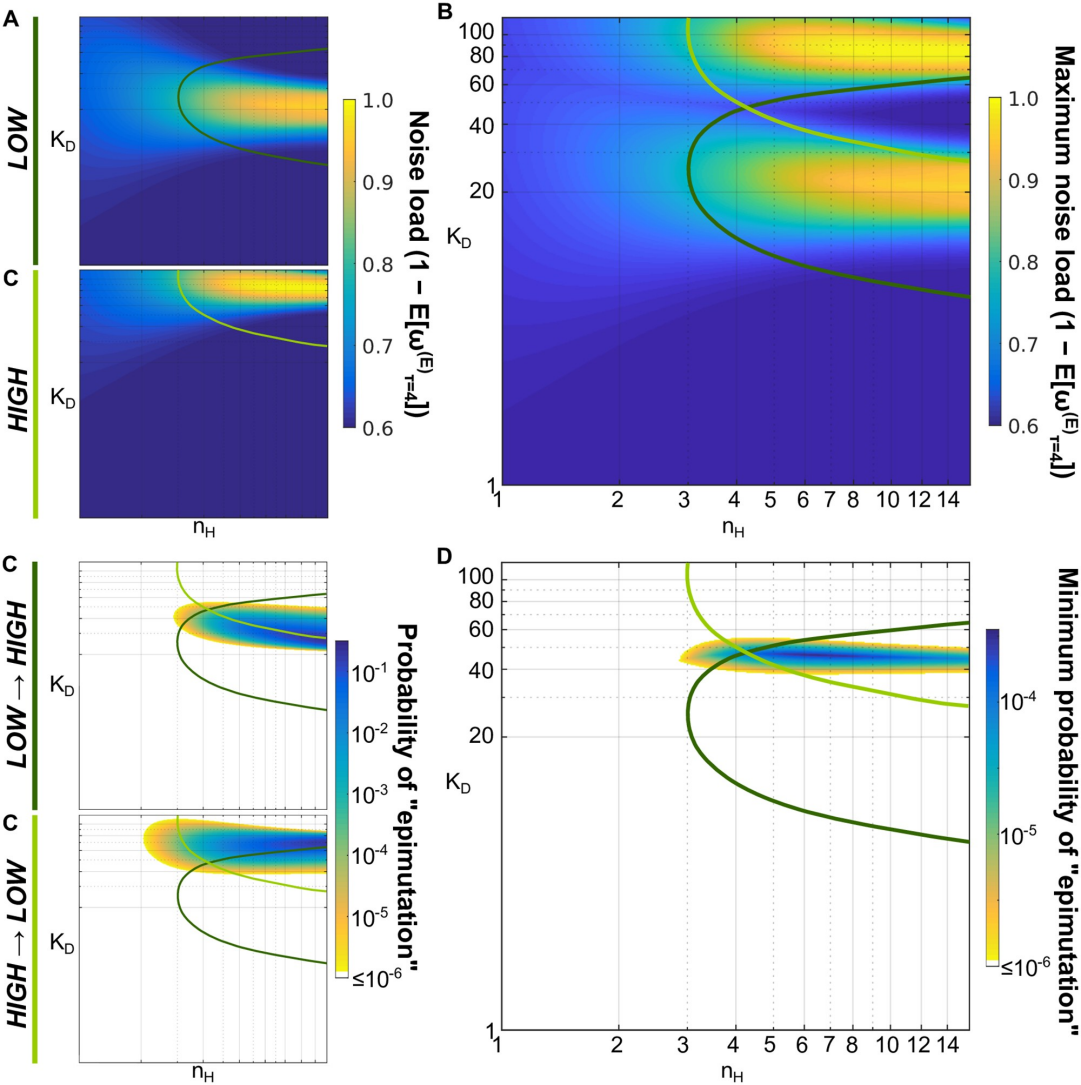
Increasing nonlinearity reduces the noise load in the bistable region, decreasing also the epimutation rate. (A) The noise load (Eq 11) is calculated for the solution genotypes $\theta_{*}^{\left(E\right)}$ for each environmental state, *LOW* and *HIGH*. (B) The maximum noise load comparing $\theta_{*}^{\left(E\right)}$ in both environmental states for each value of *n*_*H*_ and *K*_*D*_ (with $k_{*}^{\left(E\right)}$ depending on the environment being considered). (C) The probability of an individual with a solution genotype $\theta_{*}^{\left(L\right)}$ (or $\theta_{*}^{\left(H\right)}$) and initial phenotype *A*^(*L*)^ (or *A*^(*H*)^) has a good fitness score *ω* in the alternative environment at the end of the generation, i.e. *ω*^(*H*)^(*A*_*τ*=4_) ≥ 0.5 (or *ω*^(*L*)^(*A*_*τ*=4_) ≥ 0.5), which is considered as a *LOW* → *HIGH* “epimutation” (or *HIGH* → *LOW* “epimutation”). (D) The minimum probability of “epimutation” comparing $\theta_{*}^{\left(E\right)}$ in both directions, *LOW* → *HIGH* and *HIGH* → *LOW*, for each value of *n*_*H*_ and *K*_*D*_ (with $k_{*}^{\left(E\right)}$ depending on the environment being considered). Values less or equal to 10^−6^ are omitted. Same range of values and bistable region lines as in Fig 4.

The observed trade-off suggests that the cost of biochemical noise on the population plays a fundamental role in the evolutionary dynamics. For each genotype, we define the associated *noise load* as the expected decline in fitness by the end of the cell life span:

$$\begin{array}{c} E\left[1-\omega_{\tau =4}^{\left(E\right)}\right]=1-E\left[\omega^{\left(E\right)}\tau =4\right]=1-\Sigma_{a=0}^{\infty }\;\omega^{\left(E\right)}\left(a\right)\cdot \rho \left(A=a,\tau =4\right) \end{array}$$ (11)

given an optimal initial phenotype (i.e., *ρ*(*A* = *A*^(*E*)^, *τ* = 0) = 1 and $\omega_{\tau =0}^{\left(E\right)}=1$; see Methods). Noteworthy, $E[\omega_{\tau =4}^{\left(E\right)}]$ corresponds to the estimated fitness for an isogenic population after one generation in a given environment excluding the cost of genetic drift (i.e. assuming infinite population) and mutation (i.e. assuming perfect selection), then exposing the effect of the biochemical noise. In our system, we observed a significant noise load for all solution genotypes for both environments (as defined in Eq 8; Fig 7A), with an expected fitness decay of more than 60% ($E[\omega_{\tau =4}^{\left(E\right)}]\leq 0.4$, depending on the specific genotype *θ*). Moreover, around and within the bistable region –where the genetic potential is higher– the minimum noise load for each environment occurs at higher *n*_*H*_ values (Fig 7A), especially when the two environments are considered (Fig 7B). Interestingly, the noise load is lower in both environments right at the center of the bistable region, where the same genotype has steady state solutions optimal in both environments (i.e. $k_{*}^{\left(L\right)}\approx k_{*}^{\left(H\right)}$).

How can the fitness be maximized in both environments in a bistable system? Even if the associated stationary phenotype distribution (i.e. at long time scales, *ρ*(*A*, *τ* = ∞)) is bimodal and well-balanced (i.e. ∼50% of chance of being around each mode), the genotypes with high nonlinearity can display strong memory, and retain the parental phenotype for multiple generations. Increasing the memory on bistable systems increases the expected fitness once the individual displays the optimal phenotype. But this memory also directly decreases the epimutation rate (Fig 7C and 7D). Consequently, there is a fundamental trade-off between minimizing noise load and maximizing adaptation potential in bistable genotypes. Moreover, the balance between these properties depends once more on the Hill coefficient value. To further explore this theory, we run some experiments where the environment was stopped after 1,000 generations (S8 Fig). As expected, populations in constant environment still can display bistable genotypes with very high nonlinearity, resulting in low noise load and high population fitness (S8 Fig).

Similarly, this trade-off also affects the genetic adaptation strategy. In the optimal solution space, the population requires to effectively cross the bistable region by genetic mutations (usually mutations on *K*_*D*_ value for our system) to adapt genetically. The width of the bistable region increases as *n*_*H*_ increases, reducing the probability of a beneficial mutation, and then the genetic potential.

## Discussion

### Selection for higher genetic potential allows the emergence of epigenetic switching

Starting from an arbitrary initial condition and different evolutionary conditions, all populations evolved to a similar region of the genotypic space with higher fitness (see examples in Fig 3). These final genotypes often had 20 < *K*_*D*_ < 80, and large *n*_*H*_ (S3 Fig). To help understand the forces that select for large nonlinearity, we calculated the *genetic potential* for the distinct genotypes available in our gene expression model (Fig 4). We observed that both in the presence or absence of biochemical noise, all populations initially selected for high genetic potential, i.e. genotypes more sensitive to genetic mutation, allowing for efficient adaptation after each environmental transition. In our gene expression model, the genetic potential increases around the bistable region (Fig 4), which can explain the *de novo* selection or emergence of epigenetic switching starting from a non-regulated (constitutive) gene.

### Nonlinearity modulates the trade-off between adaptation potential and phenotypic robustness

The average population fitness was mostly determined by the environmental fluctuation frequency (*ν*), where fitness decreased as the epoch length decreased. This agrees with previous theoretical work, which showed that the evolutionary dynamics are governed by environmental dynamics [16, 31]. In our simulations, the correlation between fitness and epoch length arises from the balance between two competing fitness costs: reducing the time required to adapt every time the environment changes (adaptation phase; Fig 5A) and increasing the phenotypic robustness when the environment is fixed (constant phase; Fig 5B). Our work suggests that the trade-off between adaptation time and phenotypic robustness is mostly modulated by the nonlinearity level (Fig 5).

To fully understand this relationship, we estimated the *noise load*, defined here as the expected decay in the fitness arising just from the biochemical noise, for the distinct genotypes available in our gene expression model (Eq 11; Fig 7A and 7B). We show that around the region where the genetic potential is higher, the noise load is lower for higher Hill coefficient values (*n*_*H*_) regardless of the specific environmental state (LOW or HIGH), or whether the genotype is bistable. Nevertheless, in this region of the genotypic space, a fundamental trade-off exists between minimizing the noise load in a population (Fig 7A and 7B) and maximizing the adaptation potential, either through genetic adaptation (i.e. genetic potential; Fig 4) or epigenetic switching (i.e. epimutation probability; Fig 7C and 7D).

### Epigenetic switches are superior in fast fluctuating environments

Previous theoretical work established that optimal long-term growth occurs when the phenotype switching rate matches the environmental switching rate [16–19]. The phenotype could switch either due to genetic adaptation with a rate that depends on the mutation rate and mutation step-size, or due to epigenetic switching (epimutation) with a rate determined by the underlying molecular system. In the natural world, epimutation rates are often faster than genetic mutation rates [12], which suggests that fast fluctuating environments might select for epigenetic switching (ES) over genetic adaptation (GA). Previous models did not integrate and evaluate these two competing processes in a population of cells evolving in a fluctuating environment.

Our simulations confirmed that ES consistently emerges in fast fluctuating environments (Fig 6). In agreement with Soyer and colleagues [28, 37], our populations evolved to genotypes with high nonlinearity (Fig 5; S3 Fig). However, we found that bistable genotypes (ES and BA) were only favored in the presence of biochemical noise, and in CONTROL simulations only GA was observed (Fig 6C). Moreover, the trade-off highlighted above reliably predicts the conditions under which an epigenetic switch was able to outcompete genetic adaptation. The ES strategy is selected at high *ν* (when the population spends proportionally more time in the adaptation phase) because it has a faster adaptation time (Fig 5A) at the cost of lower phenotypic robustness (Fig 5B). Nevertheless, the *genetic potential* map (Fig 4) shows that GA can potentially adapt quickly (especially for larger mutation step-size *M*), but these trajectories lie in noisy regions of the genotypic space, resulting in an even higher *noise load* (Fig 7). Therefore, we conclude that ES is selected over GA in fast fluctuating environments because this strategy minimizes adaptation time through epimutation while still reducing the *noise load* in the system.

### Lineages reveal hidden selection forces & epigenetic switching persistence

All evolved bistable and monostable genotypes were relatively close to each other (Fig 6A). Our simulations had a relatively high mutation rate (*u*) such that bistable genotypes could mutate to monostable genotypes, and vice versa. The elevated rate of seeding between these subpopulations made it challenging to distinguish whether ES (bistable) was being selected for. It has been shown previously that individual history or genealogy can efficiently reveal hidden selection forces. For example, Kussell and colleagues [34, 49] demonstrated that selective pressures on a population, such as those imposed by a fluctuating environment, can be efficiently quantified by measurements on the surviving lineages. More recently, Cerulus *et al*. [50] used life-history traits of cellular growth to show that high single-cell variance in growth rate can be beneficial for the population, and that this benefit depends on the epigenetic inheritance of the growth rate between mother and daughter cells.

To this end, we analyzed the strategy of lineages across multiple cycles during our simulations. Lineage analysis demonstrated that apparent coexistence of bistable and monostable subpopulations was a transient phenomenon, and one type of strategy was typically dominant across lineages (Fig 6B and 6C). Moreover, the persistence of well-adapted lineages without genetic mutations effectively proved that epigenetic switching can outcompete genetic adaptation in fluctuating environments. Our analysis suggests that population snapshots (e.g. bimodal versus unimodal distribution of phenotypes) can miss the contribution of epigenetic switching (Fig 6D). Future experimental studies on the evolution of epigenetic switches might consider analyzing lineages using time-lapse microscopy, as done by Balaban *et al*. [13].

### Model limitations and future directions

We verified that our observations were robust to many alternative model assumptions (S9 Fig). Nevertheless, our simple model of a haploid, asexual population [51] omits some features of the evolutionary process. For example, variable population size, diploid genetics, sexual reproduction and linkage disequilibrium could all affect the evolutionary dynamics and selection of epigenetic switches in a fluctuating environment. Our model also fixed the mutation rate (*u*) and step-size (*M*), which imposes a mutational load when the population is adapted to a constant environment. Future simulations could allow natural selection to mutate and tune these parameters, which might favor genetic adaptation over epigenetic switching [52]. Our model also did not consider the case where mutations (e.g. adaptive mutation) or biophysical parameters (e.g. phenotypic plasticity) directly respond to changes in the environment. Last, we assumed that mutations continuously increase or decrease the biochemical parameters. This overlooked an important class of mutations, such as indels (i.e. rapid loss or abrupt change of function), gene duplication, and gene recruitment, which could abruptly change the topology of the gene network.

We considered the simplest genetic circuit that can exhibit epigenetic switching. However, alternative gene regulatory networks could generate different dynamics and phenotypes that are even better adapted to the fluctuating environment. For example, adding a negative feedback loop could reduce gene expression noise [53, 54] or generate oscillations [55, 56]. An oscillatory gene circuit (e.g. circadian clock) might anticipate and respond to an environment that fluctuates regularly (e.g. day/night). Future research will explore more complicated gene regulatory circuits to understand the specific environmental dynamics and evolutionary conditions that favor oscillation versus epigenetic switching in the context of genetic adaptation. This should be of broad relevance to evolutionary biologists and systems biologists.

## Methods

### Biophysical parameters

The maximum synthesis rate *k* and the degradation rate *γ* depend on time. With no loss of generality, we reduced the number of free parameters in our model by substituting time *t* with a time-dimensionless variable *τ* = *t* ⋅ *γ*. Many proteins in bacteria are not actively degraded and are diluted through cell growth and division. Thus, *τ* and *k* are in units of cell cycle time. With no loss of generality, we used *τ* = 4 in all our stochastic simulations. All mutated parameters were constrained to lie within a physiological range (10^−2^ ≤ *k* ≤ 10^3^, 10^−2^ ≤ *n*_*H*_ ≤ 16, 10^−2^ ≤ *K*_*D*_ ≤ 120) for a bacterium such as *E. coli*. The number of molecules for a transcription factor ranges between 0 − 10^3^ proteins per bacterium or concentration range 0 − 10^3^ nM for a bacterial volume of 1 fL [57, 58]. The DNA dissociation constant (*K*_*D*_) has a similar range to the underlying transcription factors [58, 59].

### Gillespie algorithm

The propensity or probability rate (*r*_*j*_) of chemical reaction *j* occurring during the next interval *dt* is related to the rates of mass-action chemical kinetics in a constant chemical reactor volume *V*. In our simple biochemical network, the propensity of protein synthesis is *f*(*A*) and the propensity of protein degradation is *γ* ⋅ *A*. Each step, given the current number of chemical species, Gillespie’s direct algorithm first calculates the propensities *r*_*j*_ and then calculates when the next reaction occurs and which one occurred. The waiting time of the next reaction is drawn from an exponential distribution with parameter *Σ*_*j*_*r*_*j*_, where the cumulative distribution function of any reaction occurring before time τ_*_ is $F\left(\tau_{*}\right)=1-e^{-\left(\Sigma_{j}r_{j}\right)\cdot \tau_{*}}$. Given that a reaction has occurred at time τ_*_, the probability that the event is reaction *i* is equal to *r*_*i*_/(*Σ*_*j*_ *r*_*j*_). Thus, for each iteration, two random numbers are required to determine τ_*_ and *i* as drawn from the probability distributions. The random numbers are generated using the “Minimal” random number generator of Park and Miller with Bays-Durham shuffle and added safeguards [60].

### Deterministic CONTROL simulations

The dynamic of the mean concentration (*A*) obeys a first-order ordinary differential equation:

$$\begin{array}{c} \frac{dA}{dt}=f\left(A\right)-\gamma \cdot A \end{array}$$ (12)

The cell life span was assumed to be long enough for steady state (where Eq 12 equals 0) to be reached before selection. We used numerical methods to calculate the steady state solution (phenotype) for any genotype and initial protein level inherited from the parent. All Gillespie, deterministic, and evolution simulations were implemented in C++ (https://github.com/mgschiavon/EpiEvoDynamics/releases), and all the analyses and figures were done using MATLAB.

### Alternative model assumptions

We tested the robustness of our results to alternative choices and assumptions in the evolutionary model.

#### Environmental fluctuations

Our fluctuations were regular and periodic with frequency *ν*. We tested whether stochastic fluctuations with frequency *ν* produced different results, even though previous work demonstrated little difference between the two types of fluctuations [5, 17, 19, 20]. Our simulations confirmed that periodic and stochastic environmental fluctuations generate the same qualitative trends (S9 Fig).

#### Selection algorithms

We used tournament selection to select the next generation of cells based on the fitness of the individuals in the current generation. Other common selection schemes are Truncation, Proportional, and Weighted selection [61]. These selection schemes produced similar results to tournament selection (S9 Fig). We also obtained similar results with a Moran model (S9 Fig), where the birth and death events are treated as continuous, stochastic events instead of non-overlapping generations (as in our modified Wright-Fisher model).

#### Fitness of phenotypes

Our simulations evaluated the protein number (phenotype) at the end of Gillespie simulation (individual life span) to calculate a fitness score given by a Lorentzian function centered at the optimal phenotype. We also used the average protein number or the distribution of protein numbers over the individual life span as phenotypes; our results did not qualitatively change (S9 Fig). We also changed the shape of the fitness function from a Lorentzian to a Gaussian or a step-like function with similar width; the results did not qualitatively change (S9 Fig).

#### Mutations

Our simulations used a spherically symmetric 3D mutation scheme to permit co-variation in biophysical parameters in a single mutational step. The mutation step size was determined by the radius of the spherical mutation, which was a uniformly distributed random value between 0 and 1 (*r* ∼ *U*(0, 1)). Such a radial density produces a non-uniform density of mutations with highest densities close to the parental phenotype because volume scales as *r*^3^. We tested homogeneous spherical mutation by substituting *r* in Eqs (5)–(7)) with $\sqrt[{3}]{r}$ and a homogeneous cubic mutation where three uniformly distributed random value between -1 and 1 (*r*_*i*_ ∼ *U*(−1, 1)) for each biophysical parameter. Both mutation schemes produced the same qualitative results (S9 Fig). We also verified that mutating only one parameter at a time (1D mutation) and increasing the range of biophysical parameters to allow higher nonlinearity (10^−2^ ≤ *n*_*H*_ ≤ 24) and weaker DNA dissociation constants (10^−2^ ≤ *K*_*D*_ ≤ 10^3^) did not fundamentally change our results.

#### Timescales of epimutation and stochastic gene expression

The rate of epimutation is sensitive to the frequency and magnitude of stochastic events. The magnitude of stochastic events is inversely proportional to the total number of molecules. Thus, we expect a higher rate of epimutation for smaller numbers of molecules. The rate of epimutation should also increase as the two modes become closer. Thus, we expect a higher rate of epimutation for larger *α*. Last, the protein degradation rate (*γ*) sets the timescale between stochastic events (i.e. faster protein degradation leads to more stochastic events per unit time during a Gillespie simulation). Thus, we expect a higher rate of epimutation for larger *γ*. In all tested cases, a higher rate of epimutation favored ES over GA (S9 Fig).

#### Matching *α* and the ratio of optimal phenotypes

At high levels of nonlinearity, the lowest protein level is *k* ⋅ *α* and the highest protein level is *k*. A bistable, epigenetic switch has two solutions, each well-adapted to one of the environments only when the ratio *R* = *A*^(*L*)^/*A*^(*H*)^ = *α* (S1 Fig). Any mismatch between *α* and *R* will disfavor epigenetic switching because an epimutation from an adapted mode will jump to a maladapted mode, after which the descendants must accumulate genetic mutations to further adapt. Although our simulations fixed *R* = *α* = 0.25, we verified that variable *α* evolved to ES with *α* ≈ *R* in fast fluctuating environments (high *ν*; S10 Fig).

### Lineage analysis

An unfit mutant could arise at the end of a cycle or a fit genotype might go extinct due to genetic drift and gene expression noise. To obtain insights on the evolutionary stability of different strategies, we focused our analysis on lineages that have persisted –with or without mutations– through at least one full environmental cycle (i.e. LOW epoch + HIGH epoch). We maintained genealogical records and traced the lineage and ancestral genotype of all cells over 2 cycles (S5 Fig). If *any* genotype between the 1-cycle and 2-cycle ancestors was monostable, then we classified the evolutionary strategy of that lineage as genetic adaptation (GA). If all genotypes between the 1-cycle and 2-cycle ancestors were bistable, then we classified the evolutionary strategy as either epigenetic switching (ES) or bistable adaptation (BA). The lineage was BA only if the 1-cycle bistable ancestor had accumulated at least one mutation since the 2-cycle bistable ancestor. At the end of each cycle, we calculated the fraction of surviving cells whose ancestors had one of these 3 strategies (GA, ES, BA) and averaged over all cycles (Fig 6). Each individual ancestral lineage was counted regardless of whether it was shared with other individuals or not.

To determine the transition rate between evolutionary strategies, we analyzed lineages back to the 3-cycle ancestor. We calculated the statistics of transitions between adaptation strategies by comparing strategies in 1-cycle and 2-cycle ancestors (current adaptation strategy) versus 2-cycle and 3-cycle ancestors (previous adaptation strategy) and averaged over all cycles (S7 Fig).

### Noise load and “epimutation” probability

The Finite State Projection (FSP) method [62] allows the estimation of a probability distribution *ρ*(*A*, *τ*) at any specific time *τ*_*f*_ having an initial distribution *ρ*(*A*, 0) and state reaction matrix **B**(*θ*):

$$\begin{array}{c} \rho \left(A,\tau_{f}\right)=\text{exp}\left(B\left(\theta \right)\cdot \tau_{f}\right)\cdot \rho \left(A,0\right). \end{array}$$ (13)

Assuming an individual with genotype *θ* was selected in the previous generation with the optimal phenotype for the environment *E*_*S*_, i.e. *ρ*(*A* = *A*^(*ES*)^, 0) = 1, *ρ*(*A*, *τ* = 4) reflects the phenotype probability distribution of the individual at the end of the following generation. This phenotype distribution can be used to calculate the expected fitness of a genotype *θ* with perfect selection and no genetic mutation, isolating the effect of biochemical noise. Then, we define the genotype’s *noise load* as the expected decay on fitness at the end of the cell life span (Eq 11; Fig 7A and 7B). Additionally, the probability that an individual selected in one environment *E*_*S*_ displays high fitness in the alternative environment *E*_*A*_ (e.g. $w_{i}^{\left(E_{A}\right)}\geq 0.5$) was used as an approximation of the epimutation probability of epigenetic switches (Fig 7C and 7D).

## Supporting information

S1 FigDeterministic steady states and stochastic stationary distributions of protein numbers given the biophysical parameters of a self-activating gene.(A) The effect of the maximum synthesis rate (*k*) and the affinity constant (*K*_*D*_) over the deterministic steady state solutions of the protein expression of a self-activating gene (i.e. $\frac{dA^{*}}{d\tau }=f\left(A^{*}\right)-A^{*}=0\Leftrightarrow f\left(A^{*}\right)=A^{*}$, where $f\left(A\right)=k(\alpha +\left(1-\alpha \right)\frac{A^{n_{H}}}{A^{n_{H}}+K_{D}^{n_{H}}})$) in the limit of high Hill coefficients (*n*_*H*_ → ∞). If *K*_*D*_ < *αk* the system is monostable HIGH with the protein expression steady state (A*) equal to *k*; on the other hand, if *K*_*D*_ > *k* then the system is monostable LOW with A* = *αk*. When *αk* ≤ *K*_*D*_ ≤ *k* is intermediate, these two steady states coexist and the system is bistable. (B) Bifurcation diagram of the protein steady states as the Hill coefficient (*n*_*H*_) varies while keeping the rest of the biophysical parameters fixed. As *n*_*H*_ value increases, the system goes from monostable (blue dots) to bistable (violet and pink dots). As *n*_*H*_ → ∞, the stable steady states monotonically approach their limiting values, *αk* and *k* (dashed gray lines), and the unstable steady state asymptotically approaches *K*_*D*_ (dotted gray line). We show a few examples of the stationary distribution of the protein expression (*ρ*(*A*, *τ* = ∞)) for stochastic simulations with intrinsic biochemical noise (bottom). As *n*_*H*_ approaches the bifurcation point (where the system passes from being monostable to bistable) the stationary distribution becomes wider (i.e. the phenotype is more variable). In the bistable region, even if the two modes of the stationary distribution do not change much, their relative weights can be significantly affected by the value of the unstable steady state, as stochastic transitions from one stable mode to the other become more or less probable.(TIF)

S2 FigGenetic potential increases around the bistable region at high nonlinearity for a toggle switch model.Here we consider an alternative model that can produce bistability and then epigenetic switching based on a toggle switch:
$$\begin{array}{ccc} \frac{dA}{dt} & = & k_{A}\cdot (\alpha_{A}+\left(1-\alpha_{A}\right)\frac{K_{A}^{n_{A}}}{K_{A}^{n_{A}}+B^{n_{A}}})-\gamma \cdot A\\ \frac{dB}{dt} & = & k_{B}\cdot (\alpha_{B}+\left(1-\alpha_{B}\right)\frac{K_{B}^{n_{B}}}{K_{B}^{n_{B}}+A^{n_{B}}})-\gamma \cdot B \end{array}$$ with *α*_*A*_ = 0.25, *k*_*B*_ = 50, *α*_*B*_ = 0.2, *K*_*B*_ = 20, *n*_*B*_ = 5, *γ* = 1, and *A* is still the molecule to be regulated and selected. In this model $f\left(A\right)=k_{A}\cdot (\alpha_{A}+\left(1-\alpha_{A}\right)\frac{K_{A}^{n_{A}}}{K_{A}^{n_{A}}+B_{*}^{n_{A}}})$, with $B_{*}=\frac{k_{B}}{\gamma }\cdot (\alpha_{B}+\left(1-\alpha_{B}\right)\frac{K_{B}^{n_{B}}}{K_{B}^{n_{B}}+A^{n_{B}}})$. Analogous to the main model (Eq 8), the solution genotype can be calculated as the values of *K*_*A*_ and *n*_*A*_ vary:
$$\begin{array}{ccc} k_{A*} & = & \frac{\gamma A_{*}}{f(A_{*})} \end{array}$$ (A) The *f*(*A*) function for some solution genotypes are shown (see row and column titles), exemplifying cases where (1) both $\theta_{*}^{\left(L\right)}$ and $\theta_{*}^{\left(H\right)}$ are monostable (i.e. the associated *f*(*A*) and *γ* ⋅ *A* intersect only once; all cases with *n*_*A*_ = 1 and {*n*_*A*_ = 16, *K*_*A*_ = 1}), (2) a bistable solution genotype with $k_{A*}^{\left(L\right)}\approx k_{A*}^{\left(H\right)}$ ({*n*_*A*_ = 16, *K*_*A*_ = 12}; *f*(*A*) and *γ* ⋅ *A* intersect both in *A* = 20 and *A* = 80), and (3) only $\theta_{*}^{\left(L\right)}$ is bistable, and not a solution for the *HIGH* environment ({*n*_*A*_ = 16, *K*_*A*_ = 44}). (B) The solution genotypes $\theta_{*}^{\left(E\right)}=\left\{k_{A*}^{\left(E\right)},n_{A},K_{A}\right\}$ per environment are shown. (C) The maximum genetic potential for different values of *K*_*A*_ and *n*_*A*_ (i.e. $\text{min}\left(d_{M}\left(\theta_{*}^{\left(L\right)}\right),d_{M}\left(\theta_{*}^{\left(H\right)}\right)\right){|}_{\left\{n_{A},K_{A}\right\}}$) is shown in the colormap. As reference, the bistable solution genotypes for each environment are delimited by the green lines: $\theta_{*}^{\left(L\right)}$ as dark green, and $\theta_{*}^{\left(H\right)}$ as light green. The colorbar shows the one-mutation distance corresponding to each value of *M*.(TIF)

S3 Fig Distribution of population average Hill coefficient values as mutation step-size *M* and environmental fluctuation frequency *ν* varies. Each line corresponds to the occurrence of the population average Hill coefficient 〈*n*_*H*_〉 in the last 5,000 generations of ten replicas of 10,000 generations simulation with evolutionary parameters: *N* = 10000, *u* = 0.03, *s*_*t*_ = 40, and *k* = 80, *n*_*H*_ = 6, and *K*_*D*_ = 45 as the initial genotype (*θ*_1_). The color determines the *M* value used, each row corresponds to different values of *ν*, and the right column shows the equivalent CONTROL simulations (i.e. without biochemical noise).
(TIF)

S4 FigIncreasing *n*_*H*_ increases the expected fitness in both environments.(A) Contour plots as a function of biophysical parameters with fixed *k* = 80 where the steady states (A*) for *LOW* are *A*^(*L*)^ ± 1% (dark green) and for *HIGH* are *A*^(*H*)^ ± 1% (light green). (B) Effect of *n*_*H*_ in the expected fitness at the end of the cell life span ($E\left[\omega_{\tau =4}^{\left(E\right)}|A_{0}=A^{\left(E\right)}\right]=\Sigma_{a=0}^{\infty }\;\omega^{\left(E\right)}\left(a\right)\cdot \rho \left(A=a,\tau =4\right)$), starting with the optimal phenotype for each environment, *k* = 80 and *K*_*D*_ as shown in the legend. In all cases, $E[\omega_{\tau =4}^{\left(E\right)}|A_{0}=A^{\left(E\right)}]$ increased as *n*_*H*_ increases. Noteworthy, for bistable solution genotypes (e.g. *k* = 80 and *K*_*D*_ = {40, 50}), $E[\omega_{\tau =4}^{\left(E\right)}|A_{0}=A^{\left(E\right)}]$ increases for both environments (even if at different rates) as *n*_*H*_ increases. The expected phenotype distribution *ρ*(*A*, *τ* = 4) was estimated numerically for each set of biophysical parameters (see Methods).(TIF)

S5 Fig Lineage analysis of cells evolving in a fluctuating environment. At the end of each cycle (LOW epoch + HIGH epoch), we analyzed the genealogy of cells over the past two cycles. All cells were classified based on the evolutionary strategy used by their 2-cycle ancestor over a full cycle (bigger dots). The top bars show the environmental state per epoch (dark green for LOW, light green for HIGH). We plot the number (#) of distinct lineages (solid line) and genotypes (dotted line) as a function of past generations on the top row. The middle rows plot the corresponding genotypes *θ* and the bottom shows the individual ancestral lineages. Ancestral genotypes can be bistable (violet) or monostable (blue). (A) Example of lineage analysis of cells that use epigenetic switching (ES) strategy for *ν* = 0.1 and *M* = 5, i.e. their 2-cycle ancestors were fully bistable and persisted a full cycle without mutations. Note that there are distinct lineages with identical genotypes. (B) Example of lineage analysis of cells that use bistable adaptation (BA) strategy for *ν* = 0.04 and *M* = 1.4, i.e. their 2-cycle ancestors were fully bistable but accumulated mutations over the next cycle. (C) Example of lineage analysis of cells that use genetic adaptation (GA) strategy for *ν* = 0.01 and *M* = 5, i.e. their 2-cycle ancestors had monostable genotypes and accumulated mutations over the next cycle. In all cases, we used *N* = 4000, *s*_*t*_ = 40, and *u* = 0.03.
(TIF)

S6 Fig Increasing selection pressure or mutation rate favors genetic adaptation, where as increasing population size favors epigenetic switching. Each colormap shows the average fraction of parental lineages using each adaptation strategy (epigenetic switching, ES; bistable adaptation, BA; genetic adaptation, GA) for the same range of mutation step-size (*M*) and environmental fluctuation frequency (*ν*) as Fig 6. Evolutionary parameters used in main text (*s*_*t*_ = 40, *N* = 10000, *u* = 0.03) are highlighted in red boxes. (A) The effect of only changing the selection pressure (*s*_*t*_) over three evolutionary replicas (*N* = 10000, *u* = 0.03). (B) The effect of only changing the population size (*N*) over three evolutionary replicas (*s*_*t*_ = 40, *u* = 0.03). (C) The effect of only changing the mutation rate (*u*) over three evolutionary replicas (*s*_*t*_ = 40, *N* = 10000). All simulations ran 10,000 generations with *k* = 80, *n*_*H*_ = 6, *K*_*D*_ = 45 as the initial genotype *θ*_1_.
(TIF)

S7 Fig Transitions between adaptation strategies as a function of evolutionary parameters. The colormaps show the percentage of ancestral lineages that displayed one adaptation strategy (current adaptation strategy) and other adaptation strategy in the preceding ancestral lineage (previous adaptation strategy). These statistics were calculated for ten evolutionary replicas for mutation step-size (*M*) and environmental fluctuation frequency (*ν*). Each simulation was run 10,000 generations with evolutionary parameters *N* = 10000, *s*_*t*_ = 40, *u* = 0.03 and *k* = 80, *n*_*H*_ = 6, and *K*_*D*_ = 45 as the initial genotype *θ*_1_. For fast fluctuating environments (large *ν*) and large mutation step size (*M*), most lineages displaying bistable adaptation (BA) as the current adaptation strategy used epigenetic switching (ES) in the previous cycle; this suggests that the stochasticity of the evolutionary dynamics is constantly feeding this subgroup. Moreover, most of the lineages using BA in the previous cycle that persisted another full cycle did it without accumulating any new mutation (i.e. using ES), suggesting the mutations occurring in the previous cycle were actually neutral. For slow fluctuating environments (small *ν*) and small mutation step size (*M*), most of the lineages used BA as the current and previous strategy, or transitioned between strategies (i.e. values not in the diagonal), suggesting the occurrence of the other strategies was just transitive, and constantly fed by the stochasticity of the process. Finally, in the “borders” between the regions where each strategy was dominant (e.g. intermediate *M* values for small *ν*), high transition rates as well as higher numbers in the diagonal occurred, congruently with the hypothesis that in these conditions the strategies have similar fitness cost and then a similar probability of being selected.
(TIF)

S8 Fig Minimizing noise load drives selection in constant environment. A simulation exemplifying the evolutionary dynamics of a population if the environment suddenly stops fluctuating. The simulation was run for 10,000 generations with evolutionary parameters *N* = 10000, *s*_*t*_ = 40, *u* = 0.03, *M* = 1.1, and *k* = 80, *n*_*H*_ = 6, and *K*_*D*_ = 45 as the initial genotype *θ*_1_; the environment fluctuates the first 1,000 generations with frequency *ν* = 0.1, and then remains constant in the LOW state (*A*^(*L*)^ = 20). (A) The population fitness (*w*) and fraction of bistable genotypes (*f*_*B*_) for each generation (*g*) are shown. Once the environment stops fluctuating (*g* ≥ 1000), *w* value increases with respect to the maximum value observed in the fluctuating environment (compared to both LOW —dark green bar— or HIGH —light green bar— environment epochs; see inset); nevertheless, once in the constant environment, *f*_*B*_ values vary widely between generations with no major effect on *w*. (B-D) Phenotype distributions for the average genotype (〈*k*〉, 〈*n*_*H*_〉, 〈*K*_*D*_〉; see boxes) in the population at several time points in the simulation (see legend) at (B) stationary state, or at the end of the life time (*τ* = 4) assuming the initial phenotype is either (C) *A*_0_ = 20 (the optimal value for the constant environment in this example) or (D) *A*_0_ = 80. In general, the population moves towards even higher nonlinearity values (*n*_*H*_) once the environment stops fluctuating, displaying sharper unimodal phenotype distributions around the optimal phenotype (C), which results in the higher population fitness *w* observed. Noteworthy, this observation holds for both monostable and bistable underlying genotypes, and regardless of the potential memory of the bistable genotypes if the initial genotype was in the alternative steady state (*A*^(*H*)^ = 80; see panel D). Similar results were obtained for different evolutionary conditions. If the population starts in a monostable genotype (*θ*_0_ = {*k* = 80, *n*_*H*_ = 1, *K*_*D*_ = 10}), as expected the population often keeps monostable genotypes faraway from the bistable region when the environment stops (particularly for small *M* values).
(TIF)

S9 Fig The same qualitative trends on the selection of adaptation strategies per evolutionary condition is maintained in a wide variety of alternative model assumptions. Each colormap shows the population average fraction of parental lineages using each adaptation strategy (epigenetic switching, ES; bistable adaptation, BA; genetic adaptation, GA) for the same range of mutation step-size (*M*) and environmental fluctuation frequency (*ν*) as in Fig 6. Differences in assumptions or parameters are listed above each plot. All values are the average of three evolutionary replicas of simulations run 10,000 generations with *N* = 4000, *s*_*t*_ = 40, *u* = 0.03 and *k* = 80, *n*_*H*_ = 6, and *K*_*D*_ = 45 as the initial genotype *θ*_1_. The exceptions are the weighted and proportional selection schemes where the selection pressure (*s*_*t*_) cannot be tuned. When the basal activity (*α*) was changed, we adjusted the low optimal phenotype such that *A*^(*L*)^ = *α* ⋅ *A*^(*H*)^, where *A*^(*H*)^ = 80. See S2 Appendix for an explicit description of assumptions or parameters.
(TIF)

S10 Fig Allowing basal activity (*α*) to evolve does not qualitatively change our results. (A) Each colormap shows the fraction of parental lineages using a specific adaptation strategy (ES, BA, or GA) averaged over all cycles and three independent replica simulations for the corresponding mutation step-size (*M*) and environmental fluctuation frequency (*ν*). Each simulation ran for 10,000 generations with evolutionary parameters *N* = 10000, *s*_*t*_ = 40, *u* = 0.03 and *k* = 80, *n*_*H*_ = 6, *K*_*D*_ = 45 and *α* = 0.25 as the initial genotype (*θ*_1_). The corresponding (B) bistable fraction (〈*f_B_*〉_sim_) and (C) basal activity parameter (〈*α*〉_sim_) averaged over all cycles and three independent replica simulations (same simulations than in panel A). For some examples, the dynamics over time for the geometric mean fitness per cycle (*W*_*cycle*_), and the average basal activity (〈*α*〉_*cycle*_), as well as the fraction of parental lineages using either ES or BA as the adaptation strategy per cycle, are shown: (D) *ν* = 0.1 and *M* = 5; (E) *ν* = 0.04 and *M* = 1.1; (F) *ν* = 0.01 and *M* = 5; and (G) *ν* = 0.01 and *M* = 1.7. When a bistable system is selected (for either ES or BA adaptation strategies), *α* ≈ 0.25. On the other hand, *α* does not show a clear selection pressure when GA is the selected adaptation strategy.
(TIF)

S1 Appendix Supplementary table: Previous works. (PDF)

S2 Appendix Alternative assumptions details. (PDF)
