# Supplementary figures and images for: Epigenetic switching as a strategy for quick adaptation while attenuating biochemical noise
Source: PLoS Comput Biol. 2019 Oct 28;15(10):e1007364. doi: 10.1371/journal.pcbi.1007364 (PMC6837633; doi:10.1371/journal.pcbi.1007364)

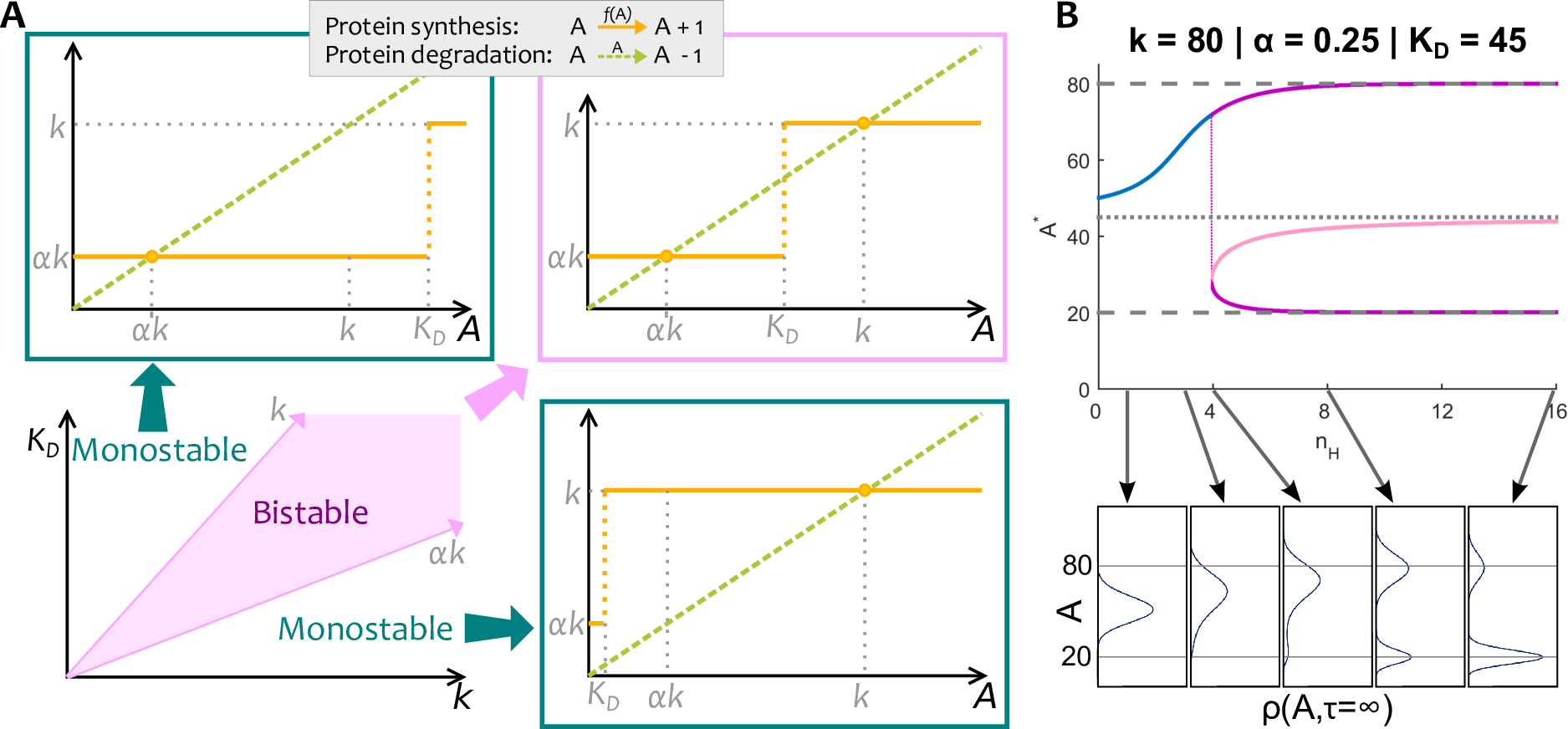

Supplement: S1 Fig — (A) The effect of the maximum synthesis rate (k) and the affinity constant (KD) over the deterministic steady state solutions of the protein expression of a self-activating gene (i.e. dA*dτ=f(A*)-A*=0⇔f(A*)=A*, where f(A)=k(α+(1-α)AnHAnH+KDnH)) in the limit of high Hill coefficients (nH → ∞). If KD < αk the system is monostable HIGH with the protein expression steady state (A*) equal to k; on the other hand, if KD > k then the system is monostable LOW with A* = αk. When αk ≤ KD ≤ k is intermediate, these two steady states coexist and the system is bistable. (B) Bifurcation diagram of the protein steady states as the Hill coefficient (nH) varies while keeping the rest of the biophysical parameters fixed. As nH value increases, the system goes from monostable (blue dots) to bistable (violet and pink dots). As nH → ∞, the stable steady states monotonically approach their limiting values, αk and k (dashed gray lines), and the unstable steady state asymptotically approaches KD (dotted gray line). We show a few examples of the stationary distribution of the protein expression (ρ(A, τ = ∞)) for stochastic simulations with intrinsic biochemical noise (bottom). As nH approaches the bifurcation point (where the system passes from being monostable to bistable) the stationary distribution becomes wider (i.e. the phenotype is more variable). In the bistable region, even if the two modes of the stationary distribution do not change much, their relative weights can be significantly affected by the value of the unstable steady state, as stochastic transitions from one stable mode to the other become more or less probable. (TIF) [file pcbi.1007364.s001.tif]

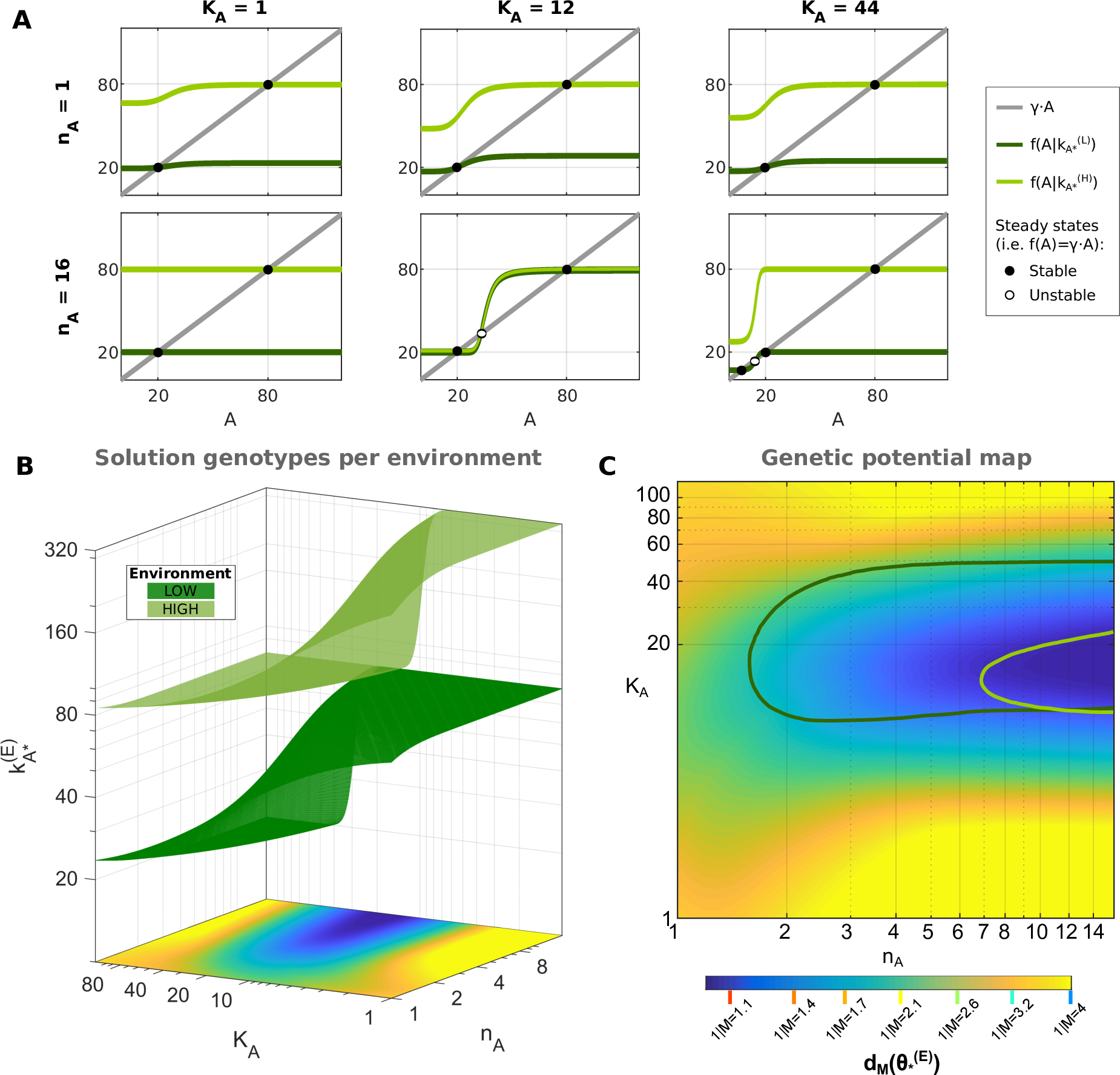

Supplement: S2 Fig — Here we consider an alternative model that can produce bistability and then epigenetic switching based on a toggle switch: dAdt=kA·(αA+(1-αA)KAnAKAnA+BnA)-γ·AdBdt=kB·(αB+(1-αB)KBnBKBnB+AnB)-γ·B with αA = 0.25, kB = 50, αB = 0.2, KB = 20, nB = 5, γ = 1, and A is still the molecule to be regulated and selected. In this model f(A)=kA·(αA+(1-αA)KAnAKAnA+B*nA), with B*=kBγ·(αB+(1-αB)KBnBKBnB+AnB). Analogous to the main model (Eq 8), the solution genotype can be calculated as the values of KA and nA vary: kA*=γA*f(A*) (A) The f(A) function for some solution genotypes are shown (see row and column titles), exemplifying cases where (1) both θ*(L) and θ*(H) are monostable (i.e. the associated f(A) and γ ⋅ A intersect only once; all cases with nA = 1 and {nA = 16, KA = 1}), (2) a bistable solution genotype with kA*(L)≈kA*(H) ({nA = 16, KA = 12}; f(A) and γ ⋅ A intersect both in A = 20 and A = 80), and (3) only θ*(L) is bistable, and not a solution for the HIGH environment ({nA = 16, KA = 44}). (B) The solution genotypes θ*(E)={kA*(E),nA,KA} per environment are shown. (C) The maximum genetic potential for different values of KA and nA (i.e. min(dM(θ*(L)),dM(θ*(H)))|{nA,KA}) is shown in the colormap. As reference, the bistable solution genotypes for each environment are delimited by the green lines: θ*(L) as dark green, and θ*(H) as light green. The colorbar shows the one-mutation distance corresponding to each value of M. (TIF) [file pcbi.1007364.s002.tif]

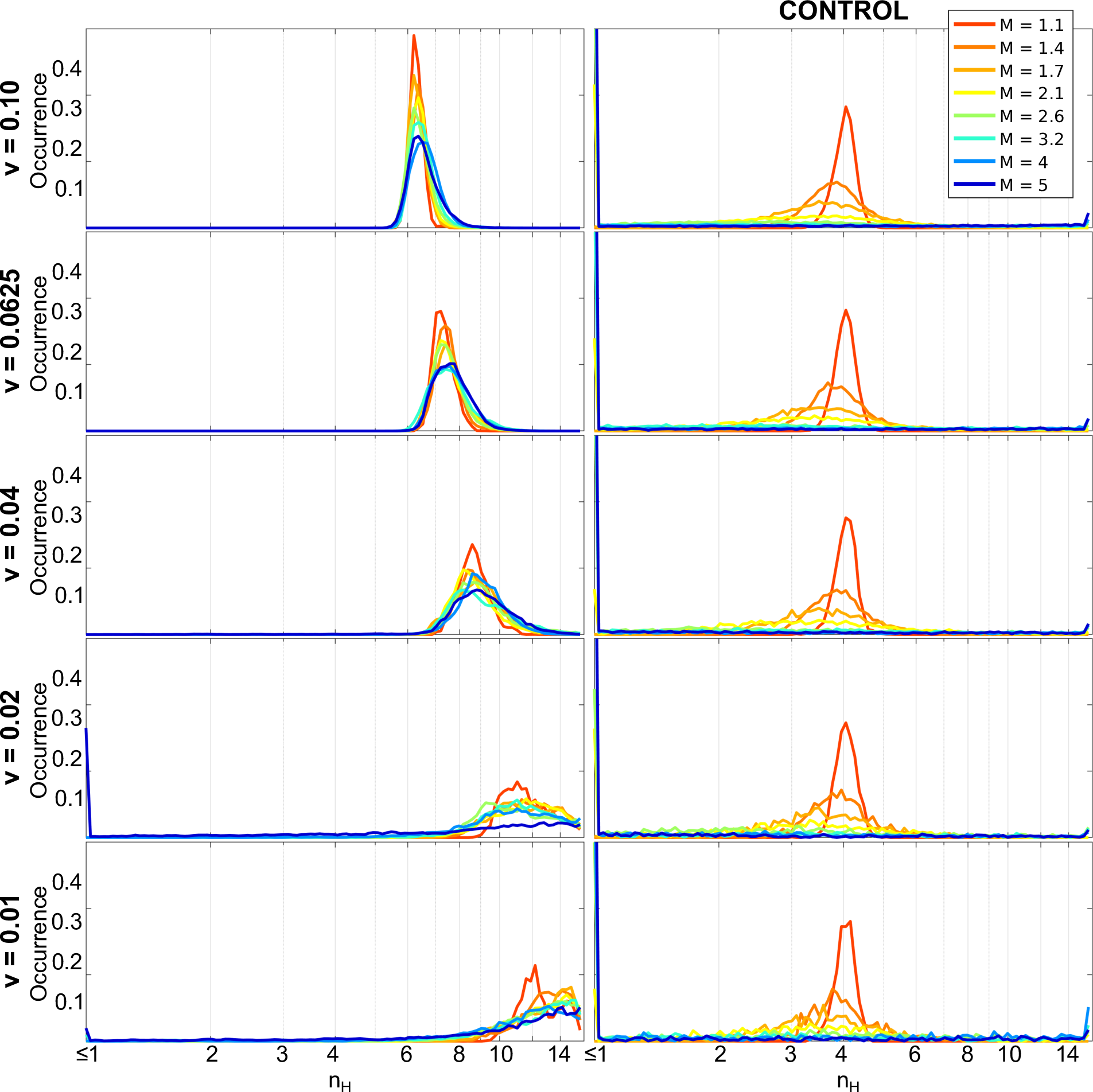

Supplement: S3 Fig — Each line corresponds to the occurrence of the population average Hill coefficient 〈nH〉 in the last 5,000 generations of ten replicas of 10,000 generations simulation with evolutionary parameters: N = 10000, u = 0.03, st = 40, and k = 80, nH = 6, and KD = 45 as the initial genotype (θ1). The color determines the M value used, each row corresponds to different values of ν, and the right column shows the equivalent CONTROL simulations (i.e. without biochemical noise). (TIF) [file pcbi.1007364.s003.tif]

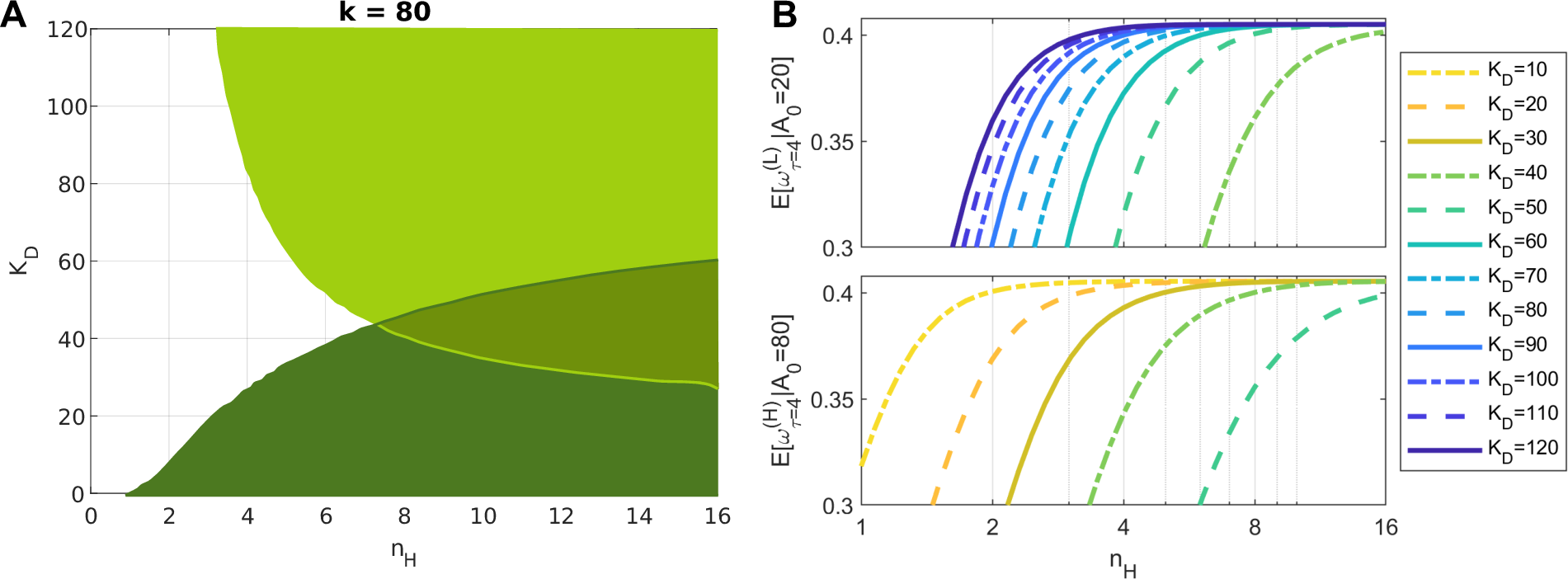

Supplement: S4 Fig — (A) Contour plots as a function of biophysical parameters with fixed k = 80 where the steady states (A*) for LOW are A(L) ± 1% (dark green) and for HIGH are A(H) ± 1% (light green). (B) Effect of nH in the expected fitness at the end of the cell life span (E[ωτ=4(E)|A0=A(E)]=Σa=0∞ω(E)(a)·ρ(A=a,τ=4)), starting with the optimal phenotype for each environment, k = 80 and KD as shown in the legend. In all cases, E[ωτ=4(E)|A0=A(E)] increased as nH increases. Noteworthy, for bistable solution genotypes (e.g. k = 80 and KD = {40, 50}), E[ωτ=4(E)|A0=A(E)] increases for both environments (even if at different rates) as nH increases. The expected phenotype distribution ρ(A, τ = 4) was estimated numerically for each set of biophysical parameters (see Methods). (TIF) [file pcbi.1007364.s004.tif]

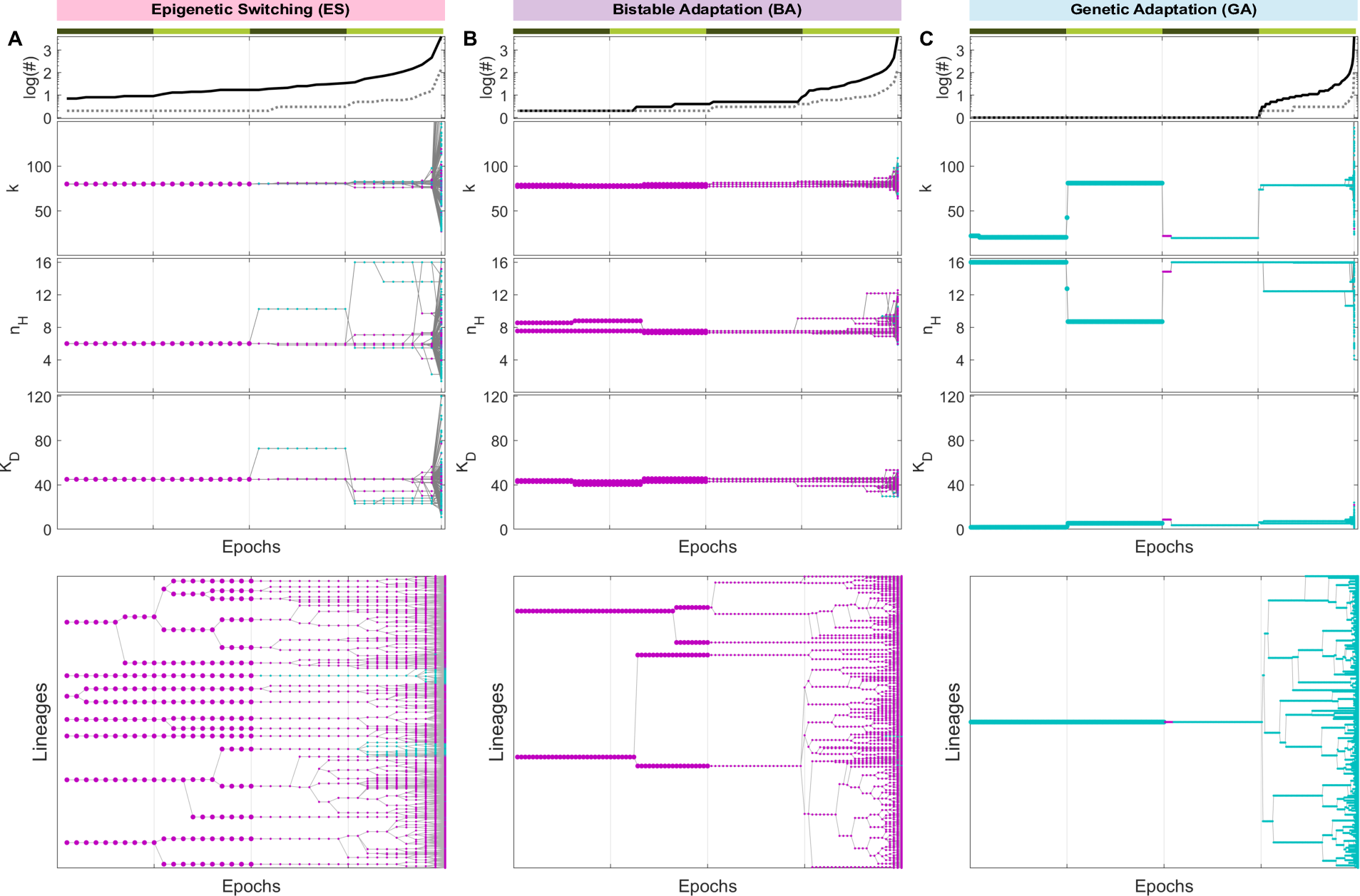

Supplement: S5 Fig — At the end of each cycle (LOW epoch + HIGH epoch), we analyzed the genealogy of cells over the past two cycles. All cells were classified based on the evolutionary strategy used by their 2-cycle ancestor over a full cycle (bigger dots). The top bars show the environmental state per epoch (dark green for LOW, light green for HIGH). We plot the number (#) of distinct lineages (solid line) and genotypes (dotted line) as a function of past generations on the top row. The middle rows plot the corresponding genotypes θ and the bottom shows the individual ancestral lineages. Ancestral genotypes can be bistable (violet) or monostable (blue). (A) Example of lineage analysis of cells that use epigenetic switching (ES) strategy for ν = 0.1 and M = 5, i.e. their 2-cycle ancestors were fully bistable and persisted a full cycle without mutations. Note that there are distinct lineages with identical genotypes. (B) Example of lineage analysis of cells that use bistable adaptation (BA) strategy for ν = 0.04 and M = 1.4, i.e. their 2-cycle ancestors were fully bistable but accumulated mutations over the next cycle. (C) Example of lineage analysis of cells that use genetic adaptation (GA) strategy for ν = 0.01 and M = 5, i.e. their 2-cycle ancestors had monostable genotypes and accumulated mutations over the next cycle. In all cases, we used N = 4000, st = 40, and u = 0.03. (TIF) [file pcbi.1007364.s005.tif]

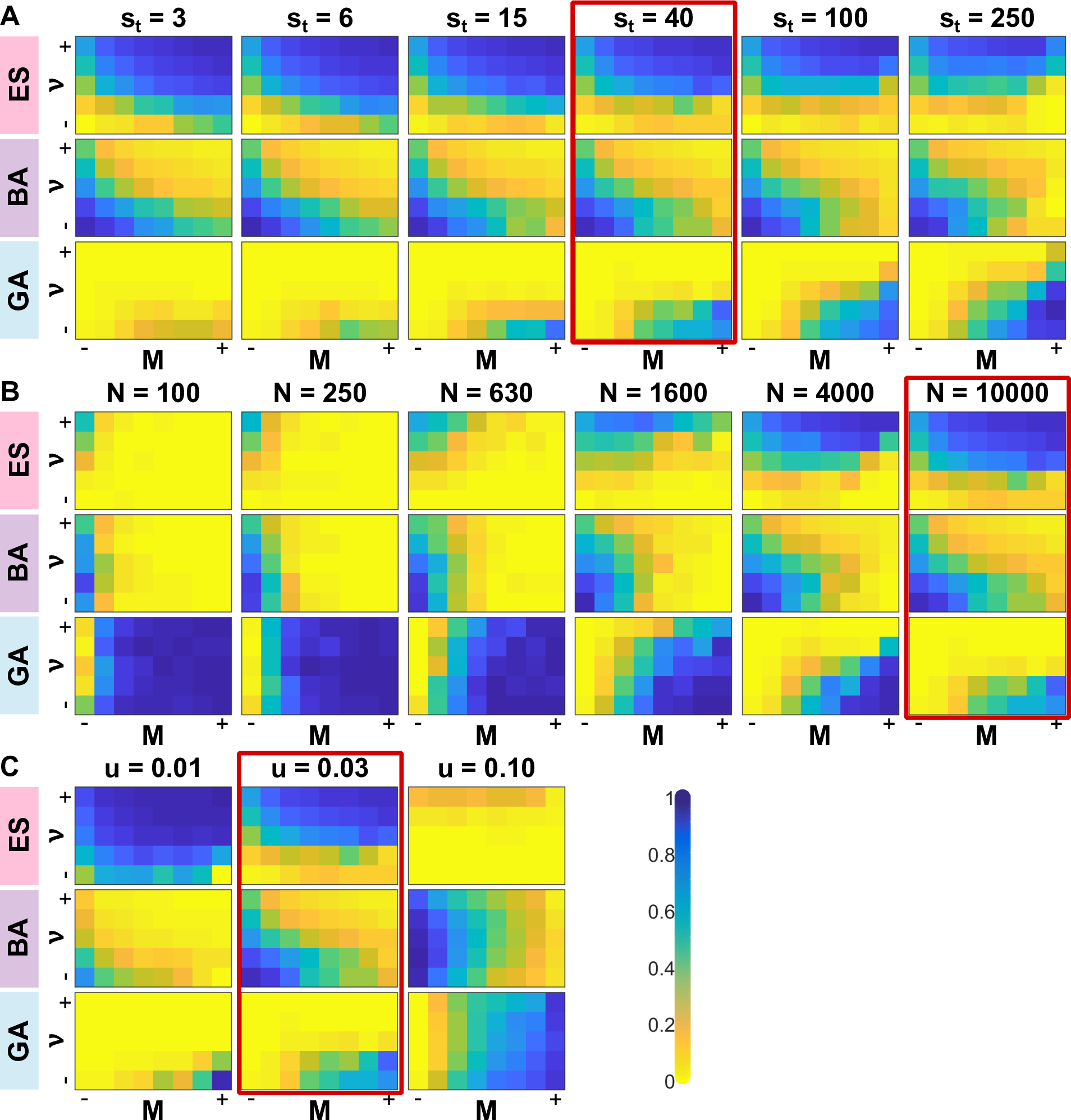

Supplement: S6 Fig — Each colormap shows the average fraction of parental lineages using each adaptation strategy (epigenetic switching, ES; bistable adaptation, BA; genetic adaptation, GA) for the same range of mutation step-size (M) and environmental fluctuation frequency (ν) as Fig 6. Evolutionary parameters used in main text (st = 40, N = 10000, u = 0.03) are highlighted in red boxes. (A) The effect of only changing the selection pressure (st) over three evolutionary replicas (N = 10000, u = 0.03). (B) The effect of only changing the population size (N) over three evolutionary replicas (st = 40, u = 0.03). (C) The effect of only changing the mutation rate (u) over three evolutionary replicas (st = 40, N = 10000). All simulations ran 10,000 generations with k = 80, nH = 6, KD = 45 as the initial genotype θ1. (TIF) [file pcbi.1007364.s006.tif]

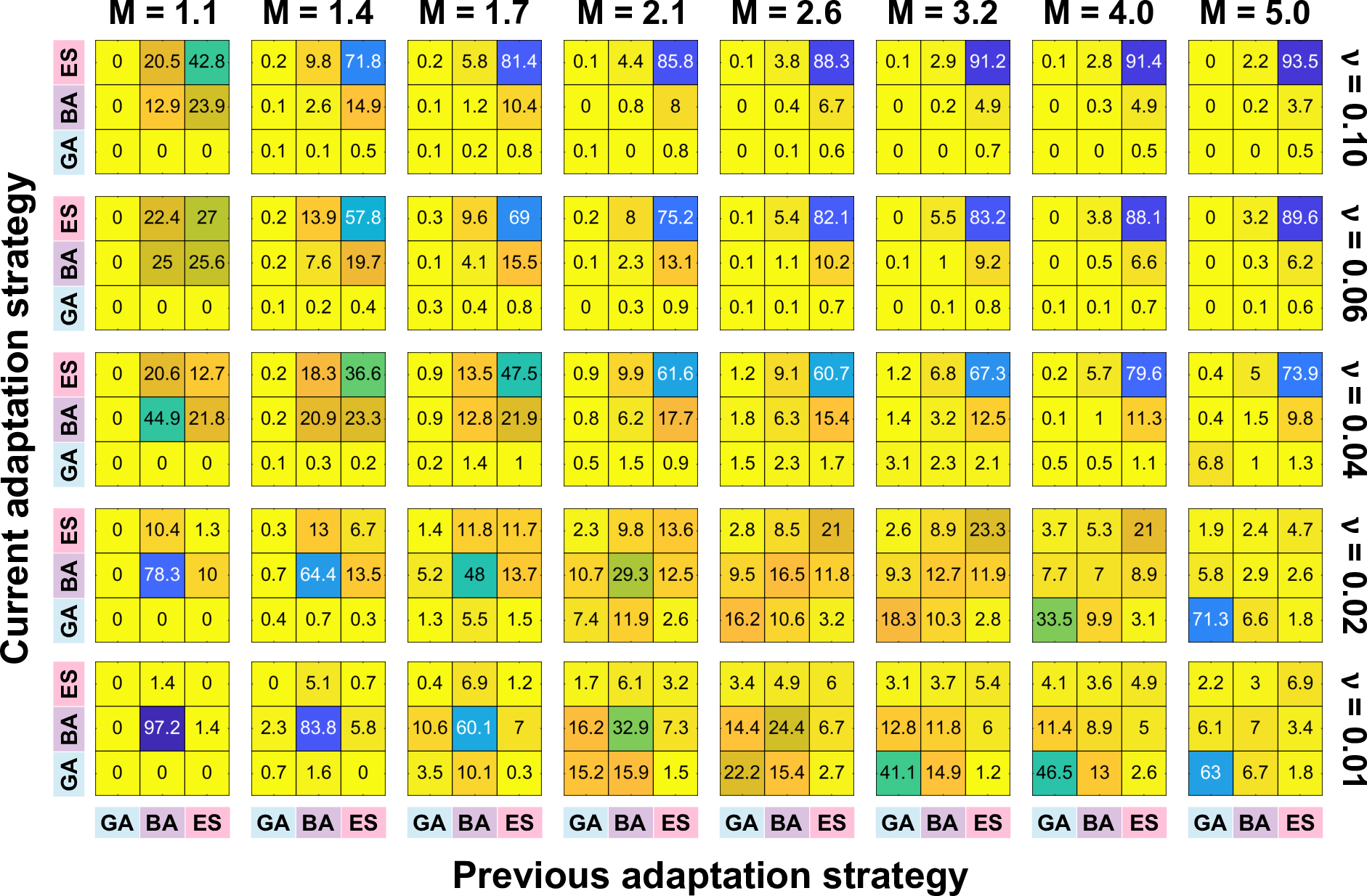

Supplement: S7 Fig — The colormaps show the percentage of ancestral lineages that displayed one adaptation strategy (current adaptation strategy) and other adaptation strategy in the preceding ancestral lineage (previous adaptation strategy). These statistics were calculated for ten evolutionary replicas for mutation step-size (M) and environmental fluctuation frequency (ν). Each simulation was run 10,000 generations with evolutionary parameters N = 10000, st = 40, u = 0.03 and k = 80, nH = 6, and KD = 45 as the initial genotype θ1. For fast fluctuating environments (large ν) and large mutation step size (M), most lineages displaying bistable adaptation (BA) as the current adaptation strategy used epigenetic switching (ES) in the previous cycle; this suggests that the stochasticity of the evolutionary dynamics is constantly feeding this subgroup. Moreover, most of the lineages using BA in the previous cycle that persisted another full cycle did it without accumulating any new mutation (i.e. using ES), suggesting the mutations occurring in the previous cycle were actually neutral. For slow fluctuating environments (small ν) and small mutation step size (M), most of the lineages used BA as the current and previous strategy, or transitioned between strategies (i.e. values not in the diagonal), suggesting the occurrence of the other strategies was just transitive, and constantly fed by the stochasticity of the process. Finally, in the “borders” between the regions where each strategy was dominant (e.g. intermediate M values for small ν), high transition rates as well as higher numbers in the diagonal occurred, congruently with the hypothesis that in these conditions the strategies have similar fitness cost and then a similar probability of being selected. (TIF) [file pcbi.1007364.s007.tif]

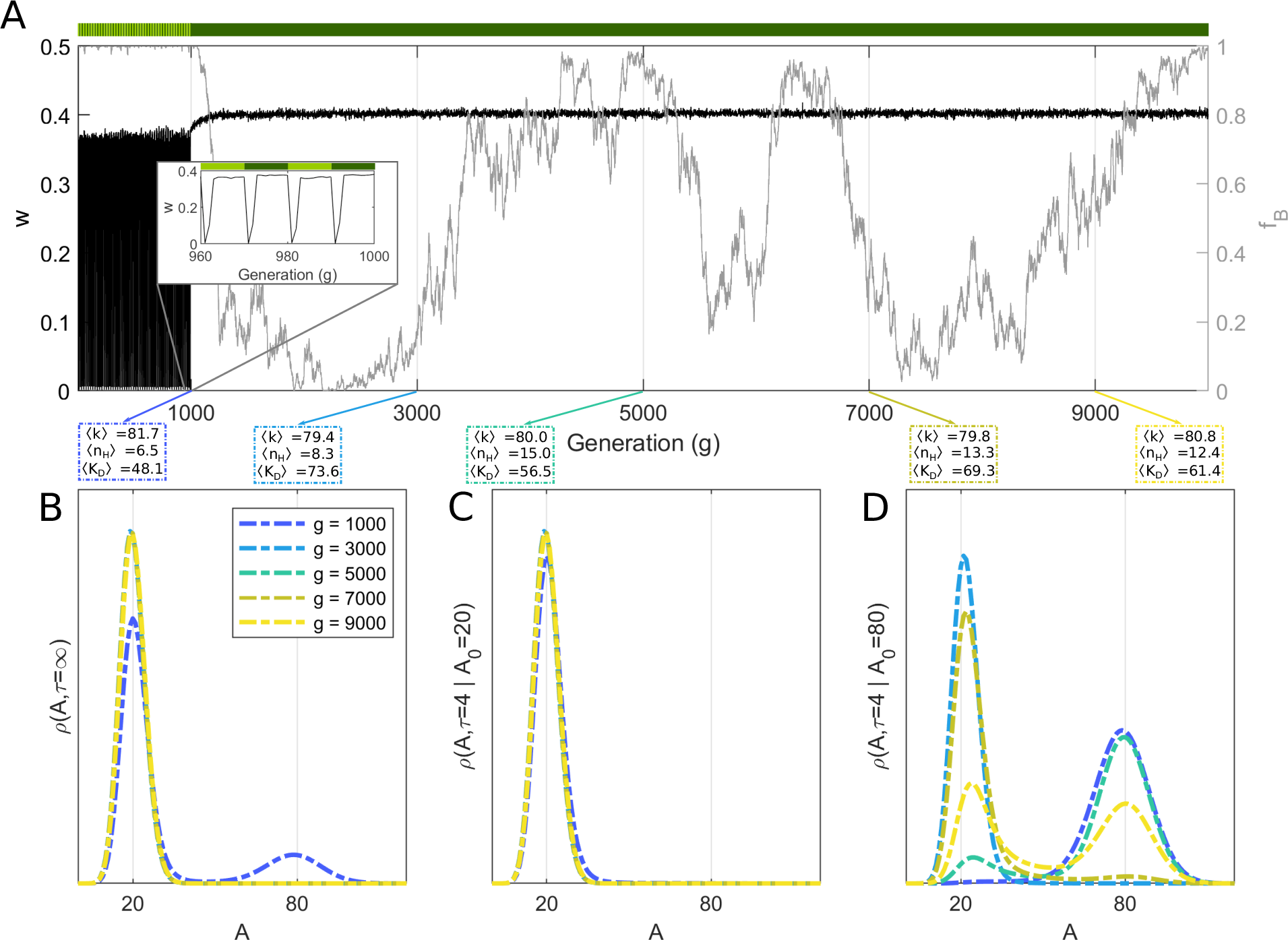

Supplement: S8 Fig — A simulation exemplifying the evolutionary dynamics of a population if the environment suddenly stops fluctuating. The simulation was run for 10,000 generations with evolutionary parameters N = 10000, st = 40, u = 0.03, M = 1.1, and k = 80, nH = 6, and KD = 45 as the initial genotype θ1; the environment fluctuates the first 1,000 generations with frequency ν = 0.1, and then remains constant in the LOW state (A(L) = 20). (A) The population fitness (w) and fraction of bistable genotypes (fB) for each generation (g) are shown. Once the environment stops fluctuating (g ≥ 1000), w value increases with respect to the maximum value observed in the fluctuating environment (compared to both LOW —dark green bar— or HIGH —light green bar— environment epochs; see inset); nevertheless, once in the constant environment, fB values vary widely between generations with no major effect on w. (B-D) Phenotype distributions for the average genotype (〈k〉, 〈nH〉, 〈KD〉; see boxes) in the population at several time points in the simulation (see legend) at (B) stationary state, or at the end of the life time (τ = 4) assuming the initial phenotype is either (C) A0 = 20 (the optimal value for the constant environment in this example) or (D) A0 = 80. In general, the population moves towards even higher nonlinearity values (nH) once the environment stops fluctuating, displaying sharper unimodal phenotype distributions around the optimal phenotype (C), which results in the higher population fitness w observed. Noteworthy, this observation holds for both monostable and bistable underlying genotypes, and regardless of the potential memory of the bistable genotypes if the initial genotype was in the alternative steady state (A(H) = 80; see panel D). Similar results were obtained for different evolutionary conditions. If the population starts in a monostable genotype (θ0 = {k = 80, nH = 1, KD = 10}), as expected the population often keeps monostable genotypes faraway from the bistable region when the [file pcbi.1007364.s008.tif]

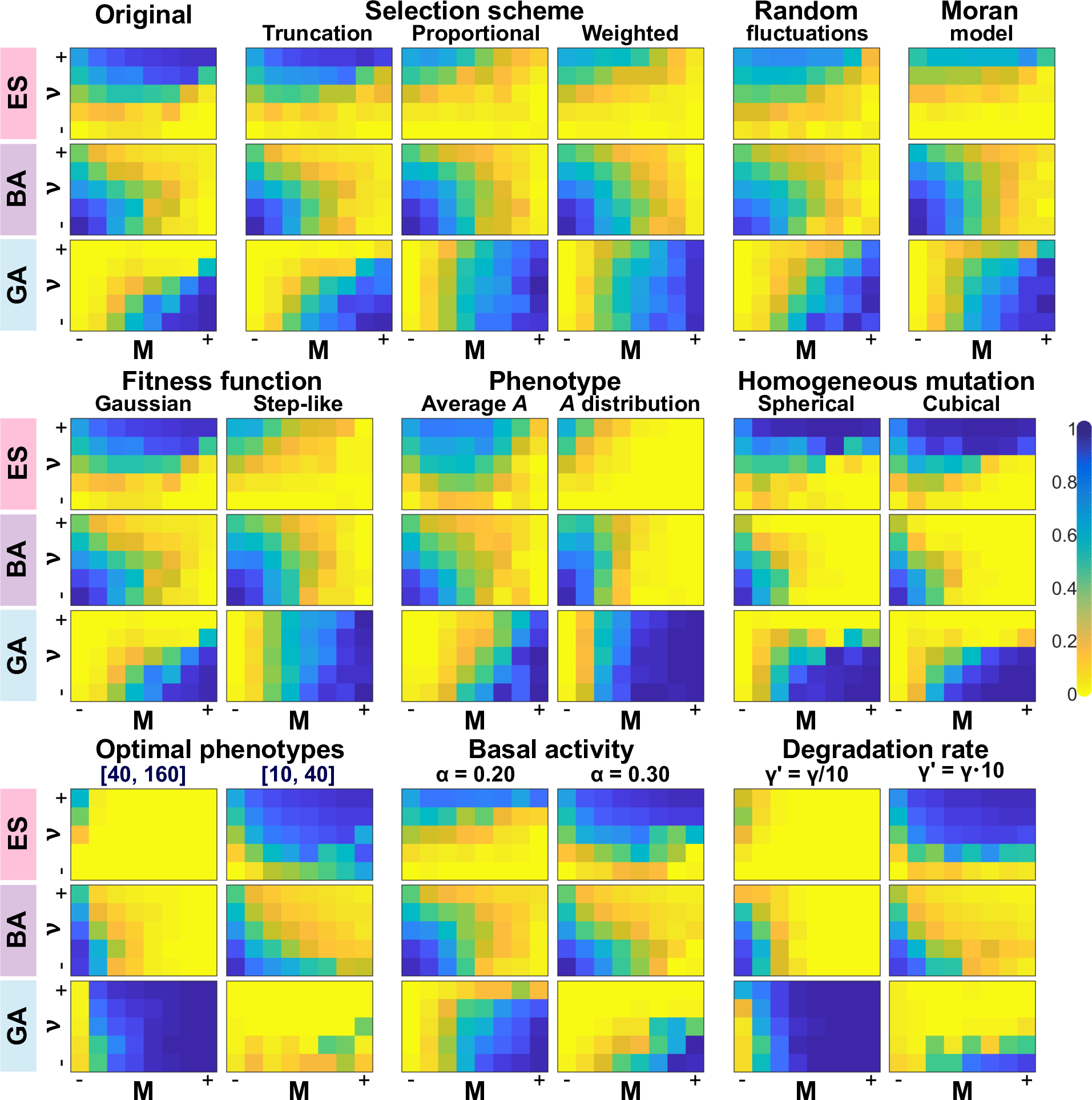

Supplement: S9 Fig — Each colormap shows the population average fraction of parental lineages using each adaptation strategy (epigenetic switching, ES; bistable adaptation, BA; genetic adaptation, GA) for the same range of mutation step-size (M) and environmental fluctuation frequency (ν) as in Fig 6. Differences in assumptions or parameters are listed above each plot. All values are the average of three evolutionary replicas of simulations run 10,000 generations with N = 4000, st = 40, u = 0.03 and k = 80, nH = 6, and KD = 45 as the initial genotype θ1. The exceptions are the weighted and proportional selection schemes where the selection pressure (st) cannot be tuned. When the basal activity (α) was changed, we adjusted the low optimal phenotype such that A(L) = α ⋅ A(H), where A(H) = 80. See S2 Appendix for an explicit description of assumptions or parameters. (TIF) [file pcbi.1007364.s009.tif]

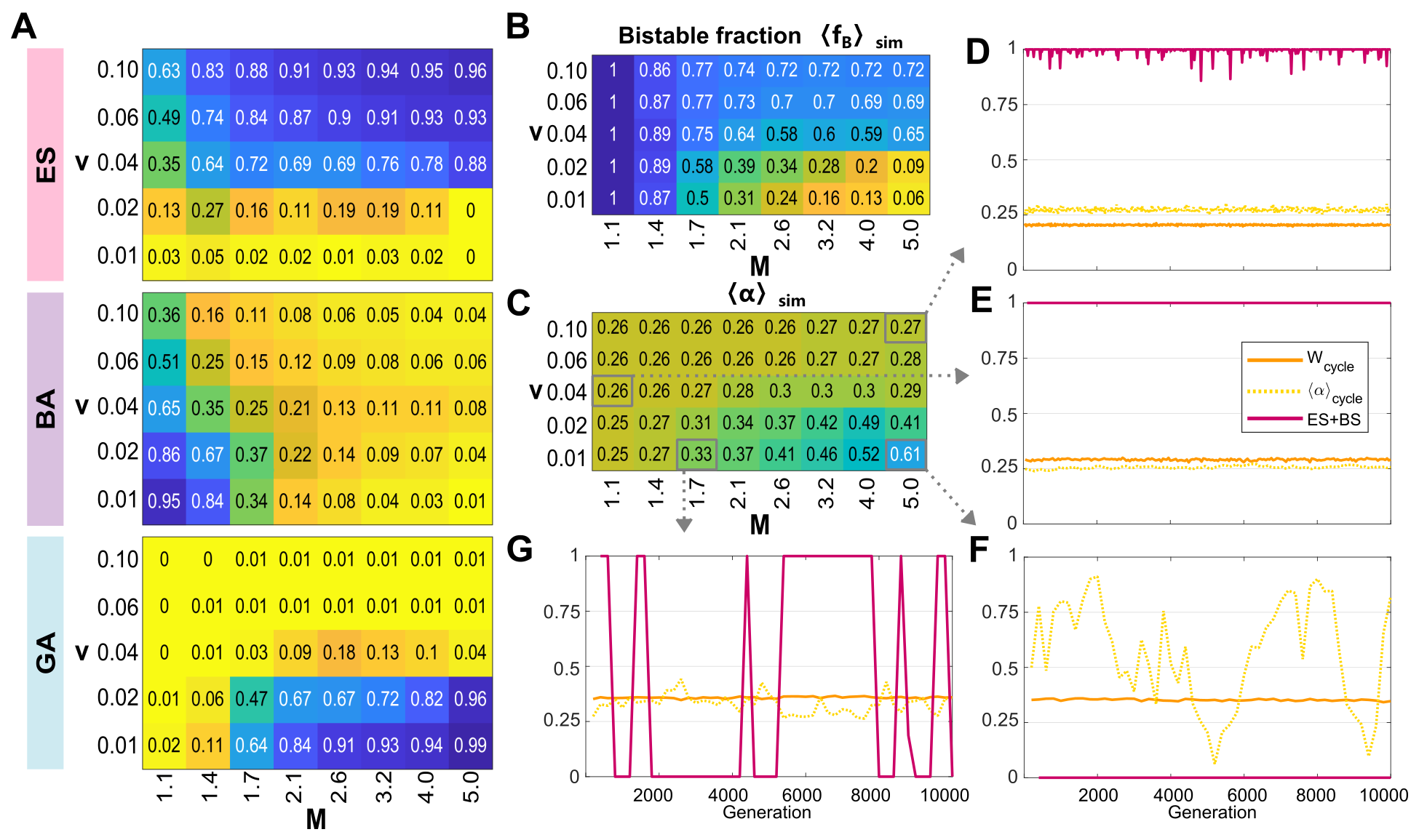

Supplement: S10 Fig — (A) Each colormap shows the fraction of parental lineages using a specific adaptation strategy (ES, BA, or GA) averaged over all cycles and three independent replica simulations for the corresponding mutation step-size (M) and environmental fluctuation frequency (ν). Each simulation ran for 10,000 generations with evolutionary parameters N = 10000, st = 40, u = 0.03 and k = 80, nH = 6, KD = 45 and α = 0.25 as the initial genotype (θ1). The corresponding (B) bistable fraction (〈fB〉sim) and (C) basal activity parameter (〈α〉sim) averaged over all cycles and three independent replica simulations (same simulations than in panel A). For some examples, the dynamics over time for the geometric mean fitness per cycle (Wcycle), and the average basal activity (〈α〉cycle), as well as the fraction of parental lineages using either ES or BA as the adaptation strategy per cycle, are shown: (D) ν = 0.1 and M = 5; (E) ν = 0.04 and M = 1.1; (F) ν = 0.01 and M = 5; and (G) ν = 0.01 and M = 1.7. When a bistable system is selected (for either ES or BA adaptation strategies), α ≈ 0.25. On the other hand, α does not show a clear selection pressure when GA is the selected adaptation strategy. (TIF) [file pcbi.1007364.s010.tif]
