# Supplementary material for: Epigenetic switching as a strategy for quick adaptation while attenuating biochemical noise
Source: PLoS Comput Biol. 2019 Oct 28;15(10):e1007364. doi: 10.1371/journal.pcbi.1007364 (PMC6837633; doi:10.1371/journal.pcbi.1007364)
Supplement: S1 Appendix — (PDF) [file pcbi.1007364.s011.pdf]

Evolutionary dynamics of an epigenetic switch in a fluctuating environment

S1 Appendix. Supplementary Table: Previous Work

Authors	Year	Evolutionary strategies	Comparison method	Analytical/ Numerical	Phenotypes	Environment	Fitness	Selection	Explicit cost	Population size	Generations	Comments
**Jablonka *et al.***	**1995**	Inducible switching; stochastic switching	Average growth rate	Both	2-discrete	Periodic & random; asymmetric (2 states)	Symmetric	Differential growth rate	Induction delay	Growing (discrete)	Non-overlapping	They compared three different strategies: non-inducible (with a small stochastic transition rate), completely inducible, or an intermediate response (memory), considering some induction delay and the phenotypic memory as a tunable property. They observed that the intermediate response is advantageous under random environmental fluctuations; if the environment is strictly periodic, the inducible system is favored unless fluctuations occur faster than the induction delay.
**Lachmann & Jablonka**	**1996**	Inducible switching; stochastic switching	Average growth rate	Analytical	2-discrete	Periodic (2 states)	Symmetric	Differential growth rate	None	Growing (discrete)	Non-overlapping	They explored the optimal values for the transition rates under fluctuating environments; they concluded that for non-inducible systems, the optimal rate for random transitions is around the frequency of the environmental fluctuations.
**Thattai & van Oudenaarden**	**2004**	Inducible switching; stochastic switching	Average growth rate	Analytical	2-discrete	Periodic & random (2 states)	Symmetric	Differential growth rate	None	Growing (continuous)	Continuous time (ODEs)	They considered that the transitions between phenotypic states depend on the environment and explored under which circumstances a transition rate to the "unfit" state different to zero will be selected; they concluded that if the transition to the "fit" state is fast enough –short induction delay–, an homogeneous population will be always favored.
**Kussell & Leibler**	**2005**	Inducible switching; stochastic switching	Average growth rate	Analytical	*n*-discrete	Random (*n* states)	No explicit assumption	Differential growth rate	Sensing, diversity & induction delay costs	Growing (continuous)	Continuous time (ODEs)	They compared inducible to stochastic transitions but taking in account the cost of sensing, the induction delay and the diversity cost imposed by the stochastic switching. They concluded that a sensor is only worth if the environment is highly uncertain, and the stochastic switching will be favored when the environment changes infrequently.
**Kussell *et al.***	**2005**	Stochastic switching	Average growth rate	Both	2-discrete	Periodic; asymmetric (2 states)	Asymmetric	Differential growth rate	None	Growing (continuous)	Continuous time (ODEs)	They considered only stochastic transitions, and they observed that the type of environmental changes determines the strategy to be used.
**Wolf *et al.***	**2005**	Fixed; inducible switching; stochastic switching	Average growth rate	Both	*n*-discrete (focus on *n*=2)	Random; asymmetric (*n* states)	Asymmetric	Differential growth rate	None	Growing (discrete)	Non-overlapping	They considered more flexible adaptation strategies, going from ignoring the environment, a deterministic inducible response, stochastic inducible response, to pure stochastic switching. If no sensor exists, stochastic switching is always selected under the time-varying environmental conditions selected here, as well as if the detection of the sensor is bad or long induction delays exist.
**Ribeiro**	**2008**	Inducible; stochastic switching (bistable genetic circuit)	Invasion	Simulations	Continuous (mechanistic)	Random (2 states)	Symmetric	Truncation selection	None	Fixed (discrete; 1000 individuals)	Non-overlapping	He modeled individual cells as toggle switches and explored the population behavior under a fluctuating environment, considering both inducible systems and pure stochastic switching. He concluded that the optimal noise level depends on the environmental fluctuations, and as noise increases, the fitness increases too in fast fluctuating environments.
**Salathé *et al.***	**2009**	Fixed; stochastic switching	Invasion	Simulations	2-discrete (x 2-modifier states)	Periodic & random (2 states)	Asymmetric	Proportional selection scheme	None	Infinite (sub-population frequencies)	Non-overlapping	Assuming an infinite population and following subpopulation frequencies through generations, they explored the impact of asymmetric fitness landscapes. They concluded that with the fitness asymmetry over a certain threshold, unless the selection pressure is very strong in both environments, ignoring the environment becomes optimal over stochastic switching (with an optimal rate approximately equal to the environmental fluctuation frequency).
**Gaál *et al.***	**2010**	Fixed; stochastic switching	Average growth rate	Analytical	2-discrete	Periodic; asymmetric (2 states)	Asymmetric	Differential growth rate	None	Infinite (sub-population frequencies)	Continuous time (ODEs)	They observed that as the asymmetry in the environments increases, the selected strategy goes from the optimal stochastic switching population (where the transition rate is assumed equal in both directions) to an equally optimal non-switching and switching populations, to finally being optimal to ignore the environment, even if a local maximum still exists for a switching rate distinct to zero.

**Visco *et al*.**	**2010**	Fixed; stochastic switching	Average growth rate	Analytical	2-discrete	Responsive (*i.e.* catastrophe rate depends on the population); random (one normal state & instantaneous catastrophe)	Asymmetric	Differential growth rate	None	Growing (continuous)	Continuous time (ODEs)	They explored the selection of stochastic switching under a single environment with occasional and instantaneous catastrophic events whose rate depends on the population structure. They observed that stochastic switching strategy is favored by strong catastrophes, while non-switching by weak catastrophes.
**Liberman *et al*.**	**2011**	Stochastic switching	Average growth rate & invasion	Both	2-discrete (x 2-modifier states with recombina-tion)	Periodic (2 states)	Symmetric	Differential growth rate	None	Infinite (sub-population frequencies)	Non-overlapping	They took Salathé *et al*. (2009) and Gaál *et al*. (2010) one step forward including recombination in the model; they observed that, under their model, recombination makes unlikely that a stable non-zero transition rate exists.
**Libby & Rainey**	**2011**	Fixed; stochastic switching	Average probability of being selected	Both	4-discrete	Periodic (one normal state & instantaneous catastrophe)	Symmetric	Strong frequency dependent selection: exclusion rule + bottleneck	Switching cost	Growing (continuous)	Continuous time (ODEs)	They considered a strong frequency-dependent selection, with an exclusion rule for the most fitted subpopulation and bottleneck when the environment changes. Even considering a switching cost –reducing the growth rate on switching genotypes–, exclusion rules are observed to favor switching phenotypes; on the other hand, larger (weaker) bottlenecks permit faster-growing, non-switching types to pass through to the next "round" outgrowing the switching type.
**Carja & Feldman**	**2012**	Stochastic switching	Probability of survival	Simulations	*n*-discrete	Periodic (2 states)	Symmetric	Proportional selection scheme	None	Fixed (discrete; 10000 individuals)	Non-overlapping	They found that phenotypic variability increases in populations under fast fluctuating environments, but this effect disappears as the fluctuations become less frequent.
**Kuwahara & Soyer**	**2012**	Genetic adaptation; stochastic switching (bistable genetic circuit)	Natural selection	Simulations	Continuous (mechanistic)	Periodic & random (2 states)	Symmetric (binary function)	Proportional selection scheme	None	Fixed (discrete; 1000 individuals)	Non-overlapping	They not only included a mechanistic model, but considered the genetic adaptation to explore the adaptive origin of stochastic epigenetic switches under fluctuating environments. They observed that bistability emerges and is maintained only in a limited range of evolutionary conditions, and suggested that its selection occurs only as a byproduct of the selection for evolvability. Noteworthy, they assumed a "binary" fitness function which would not favor the underlying bimodal distribution in a bistable system.
**Carja *et al.***	**2013**	Stochastic switching	Invasion	Both	4-discrete (x 2-modifier states with recombina-tion)	Periodic & random (*n* states)	No explicit assumption	Differential growth rate	None	Infinite (sub-population frequencies)	Non-overlapping	An extension of Liberman *et al*. (2011) model; they reached similar conclusions.
**Furrow & Feldman**	**2014**	Inducible switching; stochastic switching	Invasion	Simulations	2-discrete (x 2-modifier states with 2 epigenetic states)	Periodic & random (2 states)	Asymmetric	Differential growth rate	Epigenetic regulation	Infinite (sub-population frequencies)	Non-overlapping	They expanded the classical modifier model (e.g. Salathé *et al*., 2009) to consider inducible switching and the associated cost. They observed that the environmental fluctuation frequency influences the conditions for evolution of epigenetic regulation (either induced or stochastic switching).
**Carja *et al.* (*Genetics*)**	**2014**	Stochastic switching	Invasion	Both	2-discrete (x 2-modifier states with recombina-tion)	Periodic (2 states)	Symmetric	Differential growth rate	None	Infinite (sub-population frequencies)	Non-overlapping	They took Salathé *et al.* (2009) one step forward including migration in the model, and study the evolution of switching rates in the presence of both spatial and temporal heterogeneity in selection pressures. They observed that the evolutionary dynamics of the system are mainly governed by the environmental fluctuation rate.
**Carja *et al.* (*PNAS*)**	**2014**	Stochastic switching	Invasion	Both	4-discrete (x 2-modifier states with recombina-tion)	Periodic & random (2 states)	Asymmetric	Differential growth rate	None	Infinite (sub-population frequencies)	Non-overlapping	They took Salathé *et al*. (2009) and Liberman *et al*. (2011) one step forward including migration in the model, and compare it to the effect of mutation and recombination as sources of phenotypic variation; they observed that, under their model, these three essentially different evolutionary forces respond very similar to fluctuating selection.

**Botero *et al.***	**2015**	Inducible switching; genetic adaptation	Natural selection	Simulations	Continuous	Periodic (continuous)	Symmetric	Proportional selection scheme	Phenotypic plasticity	Fixed (discrete; 5000 individuals)	Non-overlapping	They used an abstract model which, while simple, can still display plasticity, bet-hedging, and genetic adaptation. Testing multiple environmental variation patterns, they observed that different adaptive responses consistently evolve under different timescales and predictabilities of the environmental variation.
**Lin *et al.***	**2015**	Fixed; stochastic switching	Frequency	Simulations	2-discrete	Periodic (2 states) + bottlenecks	Symmetric	Differential growth rate	None	Growing (discrete)	Continuous time	They explored the origin of the stochastic transitions in fluctuating environments distinguishing between standing variation and *de novo* mutations using both an experimental and a mathematical model. They concluded that the contribution of each of these mechanisms on the adaptation process depends on the fluctuation timescales.
**Belete & Balázsi**	**2015**	Stochastic switching	Average growth rate	Both	2-discrete	Periodic; asymmetric (2 states)	Asymmetric	Differential growth rate	None	Fixed (discrete; 10000 individuals)	Non-overlapping	They explored the stochastic switching rate dependency to the environmental fluctuation frequency in asymmetric environments and fitness as the environmental duration shorten. In this limit, they observed that the previously described optimal switching rate matching environmental fluctuation frequency does not always hold.
**Gómez-Schiavon & Buchler**	-	Genetic adaptation; stochastic switching (bistable genetic circuit)	Natural selection	Simulations	Continuous (mechanistic)	Periodic & random (2 states)	Symmetric (Lorentzian function)	Tournament selection scheme	None	Fixed (discrete; 10000 individuals)	Non-overlapping	-

Jablonka E, Oborny B, Molnar I, Kisdi E, Hofbauer J & Czaran T **(1995)**. The Adaptive Advantage of Phenotypic Memory in Changing Environments. *Philosophical Transactions of the Royal Society B: Biological Sciences, 350(1332), 133–141*.

Belete MK & Balázsi G (2015). Optimality and adaptation of phenotypically switching cells in fluctuating environments. *Physical Review E, 92(6), 62716*.

Botero CA, Weissing FJ, Wright J & Rubenstein DR **(2015)**. Evolutionary tipping points in the capacity to adapt to environmental change. *Proceedings of the National Academy of Sciences, 112(1), 184–189*.

Carja O & Feldman M (2012). An equilibrium for phenotypic variance in fluctuating environments owing to epigenetics. *J R Soc Interface, 9(August 2011), 613–623*.

Carja O, Liberman U & Feldman MW **(2013)**. Evolution with stochastic fitnesses: A role for recombination. *Theoretical Population Biology, 86, 29–42*.

Carja O, Liberman U & Feldman MW **(2014)**. Evolution in changing environments: Modifiers of mutation, recombination, and migration. *Proceedings of the National Academy of Sciences, 111(50), 17935–17940*.

Carja O, Liberman U & Feldman MW **(2014)**. The evolution of phenotypic switching in subdivided populations. *Genetics, 196(4), 1185–1197*.

Furrow RE & Feldman MW (2014). Genetic Variation and the Evolution of Epigenetic Regulation. *Evolution, 68(3), 673–683*.

Gaal B, Pitchford JW & Wood AJ **(2010)**. Exact Results for the Evolution of Stochastic Switching in Variable Asymmetric Environments. *Genetics, 184(4), 1113–1119*.

Kussell E & Leibler S (2005). Phenotypic Diversity, Population Growth, and Information in Fluctuating Environments. *Science, 309(5743), 2075–2078*.

Kussell E, Kishony R, Balaban NQ & Leibler S **(2005)**. Bacterial Persistence: A Model of Survival in Changing Environments. *Genetics, 169(4), 1807–1814*.

Kuwahara H & Soyer OS (2012). Bistability in feedback circuits as a byproduct of evolution of evolvability. *Molecular Systems Biology, 8(564), 564*.

Lachmann M & Jablonka E (1996). The Inheritance of Phenotypes: an Adaptation to Fluctuating Environments. *Journal of Theoretical Biology, 181(1), 1–9*.

Libby E & Rainey PB (2011). Exclusion rules, bottlenecks and the evolution of stochastic phenotype switching. *Proceedings. Biological Sciences / The Royal Society, 278(1724), 3574–83*.

Liberman U, Van Cleve J & Feldman MW **(2011)**. On the Evolution of Mutation in Changing Environments: Recombination and Phenotypic Switching. *Genetics, 187(3), 837–851*.

Lin WH, Rocco MJ, Bertozzi-Villa A & Kussell E **(2015)**. Populations adapt to fluctuating selection using derived and ancestral allelic diversity. *Evolution, 69(6), 1448–1460*.

Ribeiro A (2008). Dynamics and evolution of stochastic bistable gene networks with sensing in fluctuating environments. *Physical Review E, 78(6), 061902*.

Salathe M, Van Cleve J & Feldman MW **(2009)**. Evolution of Stochastic Switching Rates in Asymmetric Fitness Landscapes. *Genetics, 182(4), 1159–1164*.

Thattai M & van Oudenaarden A (2004). Stochastic Gene Expression in Fluctuating Environments. *Genetics, 167(1), 523–530*.

Visco P, Allen RJ, Majumdar SN & Evans MR **(2010)**. Switching and growth for microbial populations in catastrophic responsive environments. *Biophysical Journal, 98(7), 1099–108*.

Wolf DM, Vazirani VV & Arkin AP **(2005)**. Diversity in times of adversity: probabilistic strategies in microbial survival games. *Journal of Theoretical Biology, 234(2), 227–253*.
