# Supplementary material for: Epigenetic switching as a strategy for quick adaptation while attenuating biochemical noise
Source: PLoS Comput Biol. 2019 Oct 28;15(10):e1007364. doi: 10.1371/journal.pcbi.1007364 (PMC6837633; doi:10.1371/journal.pcbi.1007364)
Supplement: S2 Appendix — (PDF) [file pcbi.1007364.s012.pdf]

Epigenetic switching as a strategy for quick adaptation while attenuating biochemical noise

Mariana Gómez-Schiavon[1,2,3,4] and Nicolas E. Buchler[2,3,5,6]

[1] *Program in Computational Biology & Bioinformatics, Duke University, Durham, NC 27708*

[2] *Center for Genomic & Computational Biology, Duke University, Durham, NC 27710*

[3] *Department of Biology, Duke University, Durham, NC 27708*

[4] *Current Address: Department of Biochemistry and Biophysics, UCSF, San Francisco, CA, USA*

[5] *Department of Physics, Duke University, Durham, NC 27708*

[6] *Current Address: Department of Molecular Biomedical Sciences, North Carolina State University, NC, USA*

S2 APPENDIX. ALTERNATIVE ASSUMPTIONS DETAILS.

We tested the robustness of our results to alternative choices and assumptions in the presented model by: changing the used evolutionary model (Section A), allowing the environment to fluctuate randomly between the two possible states with mean frequency ν (Section B), using the average protein number or the distribution of protein numbers over the individual life span as phenotype (Section C), changing the fitness function to a Gaussian or a step-like function with similar span around the optimal phenotypes (Section D), implementing different selection schemes (Section E), as well as different mutation schemes (Section F). Additionally, more quantitative aspects of the model were perturbed by exploring other optimal phenotypes for the environments (Section G), basal activity α values (Section H), and different degradation rate γ values (Section I). In addition, we allowed the basal activity α parameter to evolve simultaneously with $\{k, n_H, K_D\}$ (see S10 Fig).

A. Moran model

Wright-Fisher and Moran models are the most common options to simulate evolution. In our main simulations, we implemented a version of the Wright-Fisher model with non-overlapping generations. Alternatively, we tested a Moran model, where the reproduction and death events are treated as stochastic events allowing overlapping generations. At each time step, an individual is chosen for reproduction using the defined tournament selection scheme, and an individual is randomly chosen from the population for death to keep the population size N fixed. N time steps occur in the previously defined lifespan time, such that the reproduction rate (and then mutation) is equivalent to the original model.

B. Environmental random fluctuations

The environmental fluctuations in our main simulations were regular and periodic with frequency ν. We tested whether stochastic fluctuations with frequency ν produced different results; even though previous work demonstrated little difference between the two types of fluctuations [1–4]. In these alternative simulations, the environment fluctuates randomly between the two possible states with mean frequency ν.

C. Phenotype definition

Our simulations evaluated the protein number (phenotype) at the end of Gillespie simulation (individual life span) to calculate a fitness score given by a Lorentzian function centered the optimal phenotype. We also tested alternative phenotype definitions: (1) the life-time average protein number to assign its fitness score to each individual in the population, or (2) the life-time protein number distribution to calculate the average fitness score for each individual in the population.

D. Fitness functions

We also changed the shape of the fitness function from a Lorentzian to a Gaussian fitness function:

$$\omega_g^{(E)}(A) = e^{-\frac{(A-A^{(E)})^2}{2\sigma^2_{(E)}}} \tag{S1}$$

where $\sigma^2_{(E)}$ is equal to the width in the Lorentzian fitness function (v^2); or a step-like function:

$$\omega_s^{(E)}(A) = \begin{cases} 1 & \text{if } (A - A^{(E)})^2 \leq 2\sigma^2_{(E)} \\ 0, & \text{otherwise} \end{cases} \tag{S2}$$

where $\sigma^2_{(E)}$ is equal to the width in the Lorentzian fitness function (v^2).

E. Selection schemes

We used Tournament selection to select the next generation of cells based on the fitness of the individuals in the current generation. Other common selection schemes are Truncation, Proportional, and Weighted selection [5].

In the truncation selection scheme, only a certain fraction of the best individuals can be selected, each with the same probability. Blickle & Thiele (1995) calculated the truncation fraction that resulted in the same selection strength as a given tournament size (Table I). They estimated that $s_t = 40$ corresponds to a 0.04 truncation fraction. We used this fraction in our Truncation selection simulation.

Using the proportional selection scheme, the probability of an individual to be selected is proportional to its fitness value. Similarly, in the weighted selection scheme, a random

TABLE I. **Evolutionary parameters**

	Parameter	Range	Units
N	Population size	$\{100, 250, 630, 1600, 4000, 10000\}$	individuals
ν	Environmental fluctuation frequency	$\{0.01, 0.02, 0.04, 0.0625, 0.10\}$	1/generation
s_t	Selection pressure (i.e. tournament size)	$\{3\,(47\%), 6\,(\mathit{24\%}), 15\,(\mathit{10\%}),$ $40\,(4\%), 100\,(\mathit{1.7\%}), 250\,(\mathit{0.7\%})\}^a$	individuals
u	Mutation rate	$\{0.01, 0.03, 0.10\}$	(1/individual) (1/generation)
M	Mutation step-size (i.e. maximum fold change)	$\{1.1, 1.4, 1.7, 2.1, 2.6, 3.2, 4.0, 5.0\}$	

[a] The equivalence between tournament size and truncation selection is shown in parenthesis. The numbers in italics were inter- and extrapolated from the values presented in [5].

individual is picked from the population and is cloned into the new population if a uniformly distributed random number (from the interval [0,1]) is below its fitness. Importantly, the selection strength cannot be directly tuned in either of these two schemes.

F. Mutation scheme

Our simulations used a spherically symmetric 3D mutation scheme to permit co-variation in biophysical parameters in a single mutational step. Co-variation is expected in a natural system as a single mutation can simultaneously affect multiple biophysical parameters; and the spherical space is a natural interpretation of M as the maximum mutation step-size, making it the maximum fold-change "distance" from the parental genotype. The actual mutation step size was determined by the radius of the spherical mutation, which was a uniformly distributed random value between 0 and 1 ($r \sim U(0, 1)$). Such a radial density produces a non-uniform density of mutations with highest densities close to the parental phenotype because volume scales as r^3. We tested homogeneous spherical mutation by substituting r in Eqs. (5-7) with $\sqrt[3]{r}$ and a homogeneous cubic mutation where three uniformly distributed random value between -1 and 1 ($r_i \sim U(-1, 1)$) for each biophysical parameter. We also verified that mutating only one parameter at a time (1D mutation) and increasing

the range of biophysical parameters to allow higher nonlinearity ($10^{-2} \leq n_H \leq 24$) and weaker DNA dissociation constants ($10^{-2} \leq K_D \leq 10^3$) did not fundamentally change our results.

G. Optimal phenotypes

The main simulations were performed with the LOW environment selecting for an optimal phenotype $A^{(L)} = 20$ proteins and HIGH environment for an optimal phenotype $A^{(H)} = 80$ proteins. The effects of doubling ($A^{(L)} = 40$ proteins, $A^{(H)} = 160$ proteins) and dividing by two ($A^{(L)} = 10$ proteins, $A^{(H)} = 40$ proteins) these values were explored.

H. Basal activity

At high levels of nonlinearity, the lowest protein level is $k \cdot \alpha$ and the highest protein level is k. A bistable, epigenetic switch has two solutions, each well-adapted to one of the environments only when the ratio $R = A^{(L)}_{opt}/A^{(H)}_{opt} = \alpha$ (S1 Fig). Any mismatch between α and R will disfavor epigenetic switching because an epimutation from an adapted mode will jump to a maladapted mode, after which the descendants must accumulate genetic mutations to further adapt. We explored the effect of other values of basal activity parameter ($\alpha = 0.2$, and $\alpha = 0.3$), but adjusting the LOW optimal phenotype accordingly ($A^{(L)} = 16$ proteins, and $A^{(L)} = 24$ proteins, respectively).

The rate of epimutation is sensitive to the frequency and magnitude of stochastic events. The magnitude of stochastic events is inversely proportional to the total number of molecules. A higher rate of epimutation for smaller numbers of molecules is expected. The rate of epimutation should also increase as the two modes become closer. Thus, we expect a higher rate of epimutation for larger α.

I. Degradation rate

The protein degradation rate (γ) sets the timescale between stochastic events (i.e. faster protein degradation leads to more stochastic events per unit time during a Gillespie simula-

tion). Thus, we expect a higher rate of epimutation for larger γ.

---

[1] Jablonka E, Oborny B, Molnar I, Kisdi E, Hofbauer J, Czaran T. The Adaptive Advantage of Phenotypic Memory in Changing Environments. Philosophical Transactions of the Royal Society B: Biological Sciences. 1995;350(1332):133–141. doi:10.1098/rstb.1995.0147.

[2] Thattai M, van Oudenaarden A. Stochastic Gene Expression in Fluctuating Environments. Genetics. 2004;167(1):523–530. doi:10.1534/genetics.167.1.523.

[3] Kussell E, Leibler S. Phenotypic Diversity, Population Growth, and Information in Fluctuating Environments. Science. 2005;309(5743):2075–2078. doi:10.1126/science.1114383.

[4] Wolf DM, Vazirani VV, Arkin AP. Diversity in times of adversity: probabilistic strategies in microbial survival games. Journal of Theoretical Biology. 2005;234(2):227–253. doi:10.1016/j.jtbi.2004.11.020.

[5] Blickle T, Thiele L. A mathematical analysis of tournament selection. In: Genetic Algorithms: Proceedings of the Sixth International Conference; 1995. p. 1–6.
